# Updates in *SASfit* for fitting analytical expressions and numerical models to small-angle scattering patterns

**DOI:** 10.1107/S1600576722009037

**Published:** 2022-11-21

**Authors:** Joachim Kohlbrecher, Ingo Breßler

**Affiliations:** a Laboratory for Neutron Scattering and Imaging, Paul Scherrer Institut, 5232 Villigen PSI, Switzerland; b BAM Federal Institute for Materials Research and Testing, 12205 Berlin, Germany; DESY, Hamburg, Germany

**Keywords:** small-angle scattering, *SASfit*, numerical models, structure factors, form factors, regularization techniques

## Abstract

Small-angle scattering is an increasingly common method for characterizing particle ensembles in a wide variety of sample types and for diverse areas of application. *SASfit* has been one of the most comprehensive and flexible curve-fitting programs for decades, with many specialized tools for various fields. Here, a selection of enhancements and additions to the *SASfit* program are presented that may be of great benefit to interested and advanced users alike: (*a*) further development of the technical basis of the program, such as new numerical algorithms currently in use, a continuous integration practice for automated building and packaging of the software, and upgrades on the plug-in system for easier adoption by third-party developers; (*b*) a selection of new form factors for anisotropic scattering patterns and updates to existing form factors to account for multiple scattering effects; (*c*) a new type of a very flexible distribution called metalog [Keelin (2016). *Decis. Anal.* **13**, 243–277], and regularization techniques such as the expectation-maximization method [Dempster *et al.* (1977). *J. R. Stat. Soc. Ser. B (Methodological)*, **39**, 1–22; Richardson (1972) *J. Opt. Soc. Am.* **62**, 55; Lucy (1974). *Astron. J.* **79**, 745; Lucy (1994). *Astron. Astrophys.* **289**, 983–994], which is compared with fits of analytical size distributions via the non-linear least-squares method; and (*d*) new structure factors, especially for ordered nano- and meso-scaled material systems, as well as the Ornstein–Zernike solver for numerical determination of particle interactions and the resulting structure factor when no analytical solution is available, with the aim of incorporating its effects into the small-angle scattering intensity model used for fitting with *SASfit*.

## Abstract 2

Recent enhancements and additions to the *SASfit* program are discussed, including anisotropic scattering models, flexible distributions, regularization techniques such as the expectation-maximization method, and new structure factors, especially for ordered nano- and meso-scaled material. The Ornstein–Zernike solver for numerical structure factors is also introduced.

## 1. Introduction

For the analysis of small-angle scattering (SAS) data, several approaches have been established in different fields of science. Typically the SAS signal is interpreted by quantities like form factor, size distribution, orientation distribution and structure factor. Depending on the pre-knowledge about the samples, different analysis techniques have been established. In cases where the investigated samples consist of dilute and identical but randomly oriented objects, like, for example, proteins, low-resolution shape reconstruction algorithms have been established (Svergun *et al.*, 1995 ▸; Svergun, 1999 ▸; Franke & Svergun, 2009 ▸; Gdovinová *et al.*, 2017 ▸; Grant, 2018 ▸, 2021 ▸; Konarev & Svergun, 2021 ▸; Schroer *et al.*, 2021 ▸). These algorithms have also been successfully used in other fields to study self-assembled structures in monodisperse colloidal systems (Luo *et al.*, 2018 ▸). Further extreme examples studied by SAS include disordered systems, which can be described by statistical means, *e.g.* using boolean models (Gille, 2016 ▸; Gommes & Roberts, 2008 ▸; Gommes, 2018 ▸) describing for instance porous media or using leveled-wave models (Berk, 1991 ▸; Ingham *et al.*, 2011 ▸; Klimeš, 2002 ▸; Jinnai *et al.*, 2000 ▸) to describe bi-continuous systems.

A significant number of scientists using SAS still describe their data very successfully by modeling them in terms of a form factor with a size distribution and, if necessary, an additional structure factor using analytical expressions (Pedersen, 1997 ▸, 2002 ▸, 2008 ▸). Several software packages for these cases are publicly available (Kohlbrecher & Studer, 2017 ▸; Breßler *et al.*, 2015 ▸; Biehl, 2019 ▸; Pedersen *et al.*, 2013 ▸; Ilavsky & Jemian, 2009 ▸; Doucet *et al.*, 2022 ▸) but only a few are referenced here. These software packages are also to some extent capable of treating textured samples where the scattering patterns are no longer radially symmetric, for example because of deformation or an orientation distribution caused by external forces like shear, or magnetic or electric fields. In cases where the internal structures of particles are of main interest, contrast-variation experiments might be necessary, or in cases where the structure factor is important, the structure-factor contribution needs to be separated from the form-factor contribution by diluting the sample. In many of these cases it is advantageous to analyze a set of several scattering patterns simultaneously with a common set of global parameters as well as a set of parameters varying from one pattern to another.

The strategy of using analytical expressions for the size distribution may limit the model too much to give a reasonable agreement with the data. To overcome this issue, regularization techniques (Lucy, 1974 ▸, 1994 ▸; Glatter, 1977 ▸; Svergun, 1992 ▸; Yang *et al.*, 2013 ▸) and Monte Carlo methods have been used (Krauthäuser *et al.*, 1996 ▸; Breßler *et al.*, 2015 ▸), mainly due to the lack of flexibility of available analytical probability distribution functions (PDFs).

Another important aspect studied by SAS is particle interaction potentials, which determine the structure-factor function. If the structure factor is the focus of a study, only a very few analytical solutions are known, such as that for hard spheres (Wertheim, 1963 ▸), sticky hard-sphere potentials (Baxter, 1968 ▸; Sharma & Sharma, 1977 ▸; Santos *et al.*, 2012 ▸, 2013 ▸) for short-range interaction potentials and a two-Yukawa potential (Hansen & Hayter, 1982 ▸; Liu *et al.*, 2005 ▸) for long-range interaction potentials. For many other interaction potentials, the Ornstein–Zernike (OZ) equation (Ornstein & Zernike, 1914 ▸; Nägele, 2004 ▸; Borowko *et al.*, 2000 ▸; Caccamo, 1996 ▸) has to be solved numerically together with a closure relation. Both need to be tested beforehand for being suitable for use with molecular dynamics (MD) simulations.

## 2. Overview

*SASfit* (Breßler *et al.*, 2015 ▸; Kohlbrecher & Studer, 2017 ▸) has been one of the most comprehensive and flexible curve-fitting programs for decades, with many specialized tools for various fields. In this article, we present major upgrades of the *SASfit* package since the last publications. We start with outlining updates on technical aspects, such as new numerical algorithms employed currently, a continuous integration (CI) practice for automated building and packaging of the software, and the upgrades on the plug-in system for easier adoption by third-party developers. Furthermore, we will focus on selected additions to *SASfit*: First, the extension of the available models of form and structure factors in *SASfit* are described in Section 4. We will also make the link between the projected correlation function measured by spin-echo small-angle neutron scattering (SESANS) and that measured by multiple SAS, and show how those models where an analytical expression for the SESANS signal exists can be efficiently used to include multiple scattering effects in SAS.

Furthermore, a new type of size distribution called the metalog distribution (Keelin, 2016 ▸) has been implemented, which was first introduced in the field of decision analysis and was designed to be a smooth PDF but also flexible enough to replace almost all known distribution functions so far. The metalog distribution can be expressed similarly to a Taylor series with any number of parameters depending on the required flexibility of the size distribution. In this article we will compare how this distribution function performs with other retrieval strategies for the size distribution.

Some newly implemented size-distribution retrieval algorithms in *SASfit* will be discussed as well, especially the expectation-maximization (EM) method introduced by Dempster *et al.* (1977 ▸), and independently by Richardson (1972 ▸) and Lucy (1974 ▸, 1994 ▸), which will be applied to SAS data. We also compare it with the strategy of fitting analytical size distributions via a non-linear least-squares fit. The EM method implementation has been extended in *SASfit* by using similar criteria to other regularization techniques to find the optimum regularization parameter. Also, a version of regularized regression using the *GSL* library (Galassi *et al.*, 2009 ▸) has been implemented for comparison.

Last but not least, a tool for numerically solving the OZ equations has been added to *SASfit*. For many models on pair interactions between colloids, no analytical solution for the structure factor is available. The OZ tool in *SASfit* now allows for a wide range of potentials and closure relations being implemented, including those trying to obtain thermodynamically consistent solutions.

## 3. Technical updates

*SASfit* is an open-source desktop application available for Windows, Linux and macOS. The computational core is implemented with the C programming language while the user interface (GUI) is realized with Tcl/Tk (Breßler *et al.*, 2015 ▸). Much progress has been achieved in the technical foundations of the program, which are summarized below.

### 3.1. Numerical algorithms

For many numerical computations, *SASfit* employs mature and reliable external third-party open-source libraries such as *GSL* (Galassi *et al.*, 2009 ▸), *FFTW* (Frigo & Johnson, 2005 ▸), *SUNDIALS* (Hindmarsh *et al.*, 2005 ▸) and *cubature* (Johnson, 2020 ▸). Third-party code is used for very efficient numerical integration algorithms in one dimension, such as the double exponential integration (Mori, 1990 ▸; Ooura & Mori, 1991 ▸; Mori & Sugihara, 2001 ▸) and specialized integration routines over the surface of a sphere [Fibonacci grid (Marques *et al.*, 2013 ▸), Lebedev quadrature (Lebedev, 1975 ▸, 1976 ▸, 1977 ▸) and spherical-*t* design (Gräf & Potts, 2011 ▸; Hardin & Sloane, 1996 ▸)] for efficient calculations of anisotropic form factors with an orientation distribution.

For numerical integrations in multiple dimensions, a more efficient and flexible routine sasf it_cubature was implemented alongside a routine for optimized spherical averages (sasf it_orient_avg). Both employ an algorithm chosen by the user in a dedicated menu, which is shown in Fig. 16 of the supporting information along with the respective parameters. The improved routines for numerical integration are now employed by models for ellipsoidal shells, triaxial ellipsoidal shells (triax ellip shell) and oriented primitive objects (OPO), as well as for orientational averages by the models for OPO. The new numerical routines result in faster calculations of the model intensities in many cases depending on the chosen algorithms. See Section 1 of the supporting information for more background on the models for OPO and the geometric primitives they describe.

The fitting of the data is carried out by a dedicated implementation of the non-linear least-squares algorithm described by Press *et al.* (1992 ▸) (Levenberg–Marquardt method). It has been extended to handle the data structures in *SASfit* and to be able to minimize multiple data sets and allow simple bounds as constraints on the fit parameters. So far no other optimization methods have been supplied.

### 3.2. Continuous integration

All source codes of the *SASfit* package are provided on the code-hosting platform GitHub (https://github.com/SASfit/SASfit) under the conditions of the General Public License (GPLv3+, https://www.gnu.org/licenses/gpl-3.0.html). The GitHub code repository is the central place for all development activities around the *SASfit* package. A CI process has been set up to automatically generate preliminary binary packages as soon as a new set of code changes is uploaded (or pushed) to the code repository. This CI process facilitates basic automated quality checks of the code, and allows interested users to try and test new models and features very early before the next full package version is released. These preliminary packages of development versions are uploaded automatically to the binary distribution platform *cloudsmith* (https://cloudsmith.io/~sasfit/repos/build/packages). All web links for downloading regular package releases and preliminary versions can be found at the project website (https://sasfit.org). It features links to the very extensive manual, which is also included in each program package, as well as additional user documentation such as video tutorials (https://www.youtube.com/@SASfitScience) and developer documentation of the source code.

### 3.3. Plug-in system

*SASfit* offers a system for grouping model functions (of form and structure factors mostly) in a plug-in. Each plug-in results in a single shared library file, which is then packaged with the program. A *SASfit* plug-in can be shared with other copies of the program of the same version on different computers. This is especially useful for customized plug-ins which users can create with the tools provided by the *SASfit* program and its source code. A typical use case is the development of a custom plug-in in the course of a research project. This plug-in could then be used with all copies of *SASfit* (of the same version) among collaborators by just copying the plug-in binary and its header file into the ‘plug-ins’ directory.

**Figure 1:**
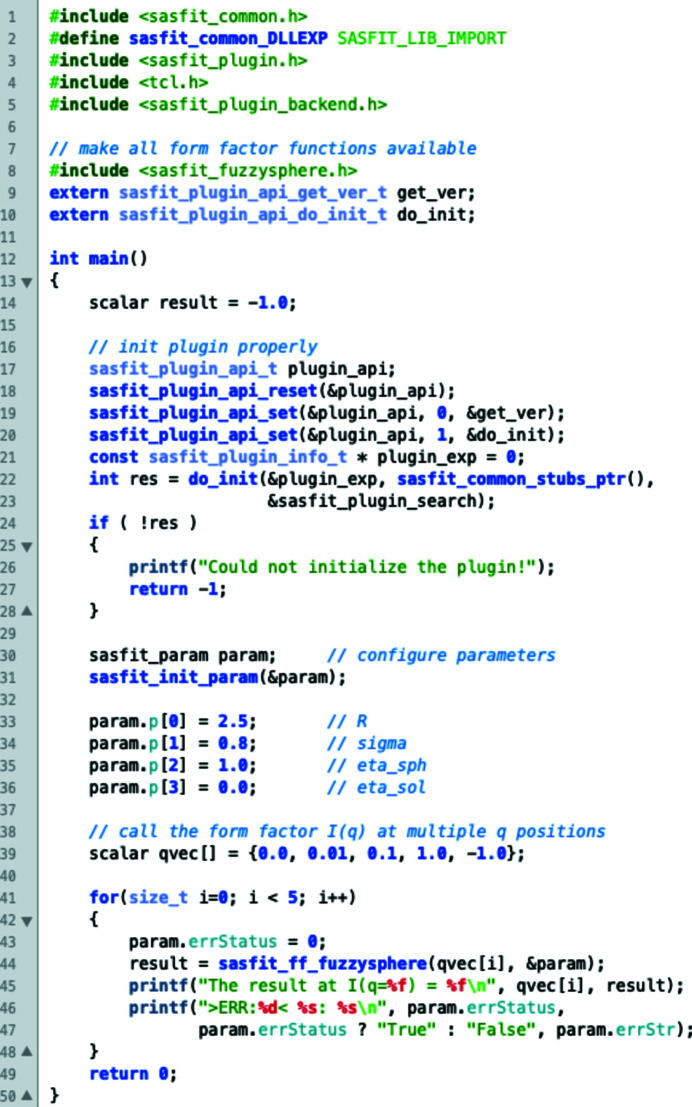
Example source code of a minimal program evaluating the *FuzzySphere* form-factor plug-in, describing scattering from spherical particles with a ‘fuzzy’ interface function.

Plug-ins can import and employ other pre-existing *SASfit* plug-ins and thus use almost the entire library of form and structure factors, currently consisting of 608 model functions in 72 plug-ins. Due to this high degree of flexibility and functional versatility, all new models added to the program are implemented as plug-ins. This large growing library of model functions might be attractive to third-party applications. To support this, source code of a minimal example of a program evaluating a single plug-in form factor (shown in Fig. 1 ▸) is provided in the main source-code tree along with documentation on how to build and run the example (https://github.com/SASfit/SASfit/tree/master/examples).

## 4. Form factors

### 4.1. Anisotropic scattering

**Figure 2:**
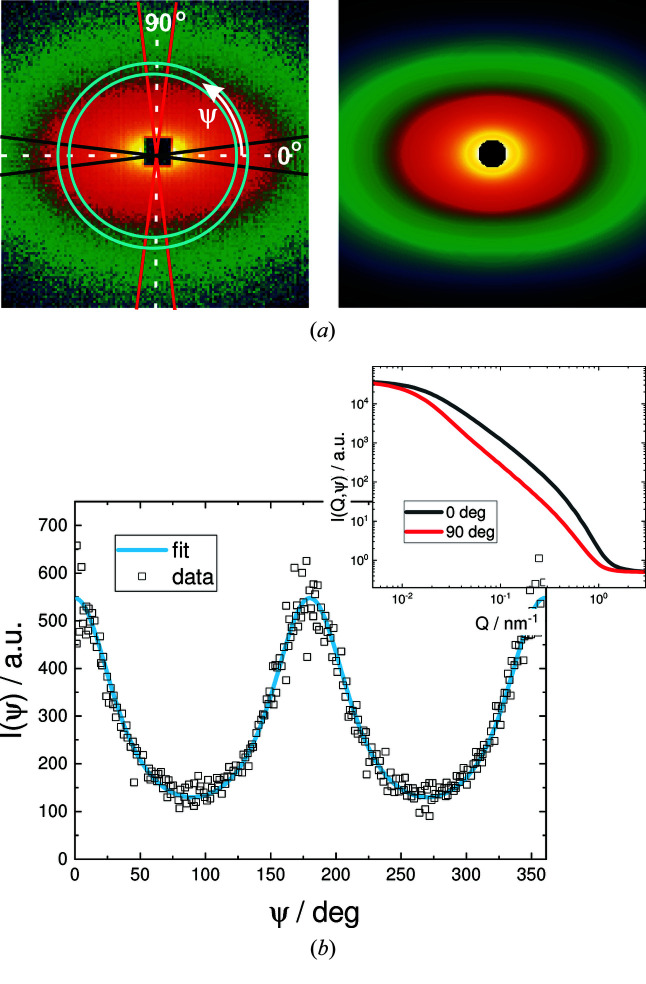
(*a*) Experimental data (top left) and 2D simulation of the fit shown in (*b*) at the same detector setting (top right). (*b*) The fit *I*(ψ) was carried out in the azimuthal direction over *Q*-averaged data points, in a small interval of *Q* within the ring shown in the graph of the experimental data (top), to obtain the orientational distribution. The structure that is scattering comprises lipid bilayers oriented in a magnetic field, which are described by discs with an orientational distribution, with details given by Liebi *et al.* (2012 ▸) and Liebi (2013 ▸). The resulting sector-averaged data *I*(*Q*, 0°) and *I*(*Q*, 90°) are shown in the inset.

In the past few years, a whole set of new form factors have been implemented in the *SASfit* package. A major part of the new form factors describe anisotropic particles with a certain orientation distribution, as well as functions describing the variation of intensity in the azimuthal (ψ) direction rather than in the radial (*q*) direction. To fit anisotropic SAS data recorded as a 2D scattering pattern, *SASfit* expects 1D input data extracted from the 2D data, either in the radial (*q*) direction from averaged sectors or in the azimuthal (ψ) direction from *Q*-averaged data points over a small interval of *Q*. *SASfit* supplies several models with an azimuthal angle ψ as an input parameter. In Fig. 2 ▸, the 2D scattering pattern of bicelles decorated with lanthanide complexes aligned in a magnetic field is shown (Liebi *et al.*, 2012 ▸; Liebi, 2013 ▸). To fit the scattering pattern, either several radially averaged sectors in certain directions ψ are taken, which can then be fitted simultaneously, or an azimuthal intensity is extracted from the 2D plot to be fitted as in this case. Here, the azimuthal fit was sufficient as the other geometrical parameters of the bicelles could be extracted in zero field from isotropic spherically averaged data. Most anisotropic models are implemented twice as a function of *Q* for a fixed value of ψ to fit sector-averaged data or *vice versa* to allow for fitting in the azimuthal direction.

### 4.2. Multiple scattering

For a small number of form factors the contributions of multiple scattering effects can be calculated, namely for monodisperse and lognormal-distributed polydisperse spheres, the Debye–Anderson–Brumberger (DAB) model (Debye *et al.*, 1957 ▸; Debye & Bueche, 1949 ▸) and the generalized Gaussian coil model, to be found under the form-factor menu ‘by plugins’ → ‘MSAS’. *SASfit* uses a formalism introduced by Schelten & Schmatz (1980 ▸) and Jensen & Barker (2018 ▸). They have shown that a multiple SAS signal can be computed from a single scattering approximation (the scattering of the sample volume in the absence of multiple scattering) via an intermediate function *i*_1_(*r*): 

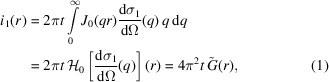




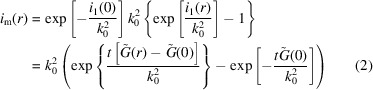

and 

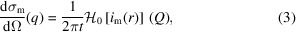

with 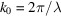 , *J*_0_ being the Bessel function of the first kind and zero order, and 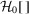 being the Hankel transform operator. Further parameters include the sample thickness *t* and the wavelength λ. 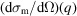 is the measured scattering cross section, including multiple scattering contributions normalized on the sample volume and corrected for absorption and incoherent scattering, *i.e.* corrected for all beam attenuation effects except coherent SAS. 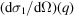 is the corresponding single scattering cross section per volume. The intermediate function *i*_1_(*r*) is mostly identical to the projected correlation function 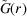 used in the theory of SESANS or spin-echo modulation SANS analysis except for a pre-factor (Kohlbrecher & Studer, 2017 ▸):

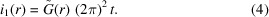

In principle the formalism in equations (1) [Disp-formula fd2]–(3) allows one to include multiple scattering effects for any kind of model with radially symmetric scattering (isotropic models), and the models have to decay faster than *q* ^−2^ otherwise the Hankel transform diverges. To calculate the projected correlation function, the Hankel transform is already implemented in *SASfit* to be applied on any available model function for 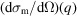 (Kohlbrecher & Studer, 2017 ▸) and can be chosen in the ‘transform’ selection box in the bottom-right corner of the model parameter window, next to the progress bar. However, the multiple scattering contribution consists of one integral for the size distribution, one integral for the Hankel transform and the subsequent backward Hankel transform with another numerical integration. These three subsequent numerical integrations slow down the numerical calculation too much to be used efficiently. Therefore, at the moment, only the analytical models for projected correlation functions are made available as scattering curves including multiple scattering effects because the forward Hankel transform is known analytically and only two integrals need to be calculated in this case. To include multiple scattering effects, the scale parameter will be the total scattering cross section per sample volume, 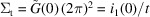 , for all models. Furthermore, all respective models contain two parameters depending on the experimental conditions, which are the used wavelength λ and the sample thickness *t*. Similarly to the arguments for SESANS, the units should be chosen so that λ has the reciprocal units of the scattering vector *q*, *i.e.* nm or Å, and the thickness should have the reciprocal units of the scattering cross section per volume, which is normally supplied in units of cm^−1^. By this parametrization, the intermediate function *i*_m_(*r*) reads as 

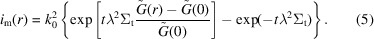

**Figure 3:**
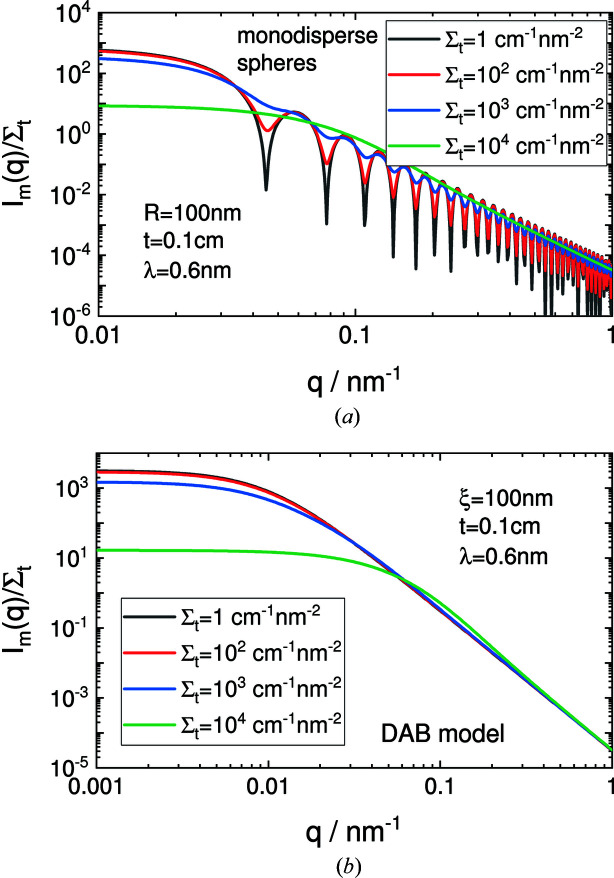
Multiple scattering effects of (*a*) monodisperse spheres and (*b*) the DAB model for total scattering cross sections Σ_t_ between 1 and 10^4^ cm^−1^ nm^−2^, a sample thickness of 0.1 cm, and a wavelength of 0.6 nm. The DAB model calculates the scattering of a randomly distributed (*i.e.* non-particulate) two-phase system assuming sharp interfaces between the phases. The two-phase system is characterized by a single length scale, the correlation length ξ, which is a measure of the average spacing between regions of phase 1 and phase 2.

Including multiple scattering in the data analysis means that, next to material properties, instrumental parameters such as sample thickness and the wavelength used also need to be known. The influence of multiple scattering is shown in Fig. 3 ▸ for monodisperse spheres and the DAB model (Debye *et al.*, 1957 ▸; Debye & Bueche, 1949 ▸). For these two models, the projected correlation function can be calculated analytically. For the more general case of multiple scattering, the package *MuScatt* (Jaksch *et al.*, 2021 ▸) might be an option.

## 5. Size distributions

### 5.1. The metalog distribution

Retrieving size-distribution information from SAS data is a standard task. Typically, two strategies are used for it: One is to model the size distribution by a known analytical expression. This is actually the main strategy followed by the *SASfit* package so far. The drawback of this method is that the solution is constrained by the shape of the chosen distribution function. Using analytical expressions for the size distribution has suffered so far from the lack of flexibility of the available distribution functions. Many distribution functions have two or three parameters for location, skewness and sometimes kurtosis, which can be expressed in terms of moments of the PDF. So far, in cases of more advanced distributions, a sum of multiple distributions was the only way to describe them.

**Figure 4:**
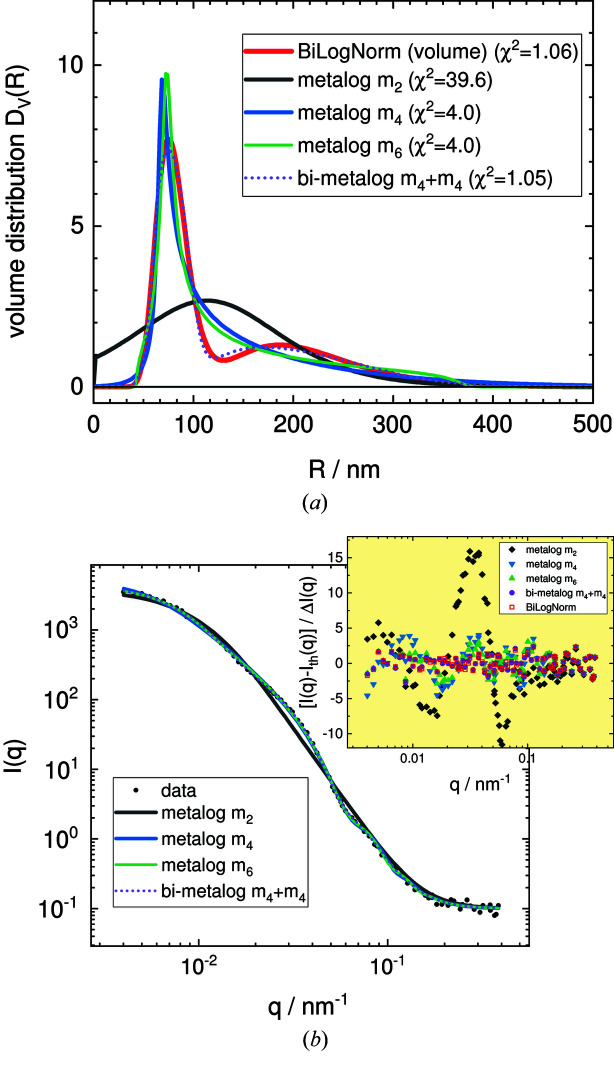
(*a*) Examples of the metalog radius distribution with two (black line), four (blue line) or six terms (green line), a bimodal metalog variant of 2 × 4 terms (dashed line), and the bimodal lognormal distribution (red line). (*b*) The resulting scattering intensities. Black circles represent the original data, and the bimodal lognormal distribution is omitted because it overlays the bimodal metalog variant exactly, as can be seen in the residual plot (inset). The original data comprise a simulated scattering curve of a bimodal distribution with Gaussian noise added, which was introduced for benchmarking by Jemian (2013 ▸) (file data/test.sas).

A newly introduced distribution function, called the metalog distribution, tries to overcome the limitation in shape flexibility. The distribution has been introduced in the area of decision analysis (Keelin & Powley, 2011 ▸; Keelin, 2016 ▸, 2021 ▸; Wikipedia Contributors, 2021 ▸; Powley, 2013 ▸). In this approach, the cumulative distribution function (CDF) rather than the PDF is directly fitted to the data. The derivative of the CDF results in the PDF. The metalog distribution can be expressed in a similar manner to a Taylor series, with any number of terms depending on the required degree of shape flexibility. It has been shown by Keelin (2016 ▸) that a ten-term metalog distribution is capable of reproducing shapes almost identical to a large number of distributions, including normal, log­normal, Weibull, gamma, exponential, chi-squared, extreme-value, beta, uniform and triangular distribution. We have implemented this distribution with a maximum of ten terms in *SASfit* to allow more flexibility in determining the shape of a size distribution. Example plots of metalog distributions with two, four or six terms mimicking a bilognormal distribution and the resulting scattering intensities are shown in Fig. 4 ▸. Even though the metalog distribution is quite flexible in its shape and can reproduce a large number of distributions, it remains smooth and even a fit with several parameters does not become so ill posed that it yields unstable solutions. The metalog distribution might be a useful choice if, next to the mean and width, some information about the skewness and kurtosis are also extractable from the SAS data.

The metalog distribution is defined via its quantile probability distribution *Q*(*y*) (QPD). The QPD in statistics is the inverse CDF. In the calculation of SAS data, one needs to integrate the form factor in the case of a size distribution over a probability density function *p*(*x*) representing the size distribution, *i.e.* the integration is carried out over the random variable *x*. The metalog distribution is, however, defined via its CDF: 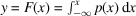 with *y* ∈ [0, 1]. The inverse of this function is the QPD: *x* = *Q*(*y*) = *F* ^−1^(*y*). Differentiating the QPD with respect to *y* yields the quantile density function *q*(*y*) = d*x*/d*y* = d*Q*(*y*)/d*y*, whose reciprocal defines the probability density function *p*(*x*) = 1/*q*(*y*) = *m*_*k*_(*y*). The metalog PDF *m*_*k*_(*y*) is therefore parameterized in terms of *y* instead of *x*. The QPD of the metalog distribution *Q*(*y*) = *M*_*k*_(*y*) [according to Keelin (2016 ▸)] and its PDF are given in the supporting information [equations (26) to (31)] for an unbounded distribution as well as distributions that are left bounded and bounded from both sides.

The integration of a form factor *P*(*Q*, *x*) over one of its size parameters *x* can be written as 

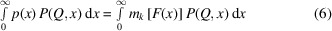

and 

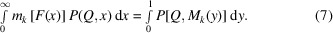

The last equation is obtained by a change of variables using d*x*/d*y* = d*M*_*k*_(*y*)/d*y* = 1/*m*_*k*_(*y*). The change of variable has the additional side effect that now the integration over the size distribution becomes an integral with finite limits over the cumulative distribution *y* from 0 to 1, which behaves numerically well for both very sharp and very broad distributions.

### 5.2. Regularization techniques

The second strategy for obtaining size-distribution information is to employ regularization techniques, which determine a model-independent distribution function. Regularization techniques are required as retrieving the size distribution from a scattering experiment is an ill-posed problem.

*SASfit* implements a standard Tikhonov regularization technique (Tikhonov, 1943 ▸; Tikhonov *et al.*, 1995 ▸) with a cost function, which can be chosen to be either an identity operator or a first- or second-order derivative operator. The Tikhonov regularization and the optimization of the weighting factor for the cost function have been implemented using standard functions provided by the *GSL* library (Galassi *et al.*, 2009 ▸).

An entropy cost function is also statistically very well supported, *i.e.* the maximum-entropy method (MEM) (Skilling & Bryan, 1984 ▸; Hansen & Pedersen, 1991 ▸; Elliott & Hanna, 1999 ▸; Hansen, 2000 ▸; Vestergaard & Hansen, 2006 ▸). As the cost function becomes non-linear, the minimization algorithms get a bit slower compared with a linear cost function in the Tikhonov regularization, where linear regression tools can be used. In *SASfit*, for the MEM, an iterative scheme has been implemented called EM, which was first explained by Dempster *et al.* (1977 ▸). The method is an iterative fixed point method for positive defined functions. Vardi & Lee (1993 ▸) have shown how the EM method can be applied to solve Fredholm integrals for the domain of non-negative real valued functions. The smearing of a form factor by a size distribution belongs to that class of Fredholm integral. The method described there is equivalent to the Lucy–Richardson method (Richardson, 1972 ▸; Lucy, 1974 ▸). The method has been applied for calculating a size distribution from scattering data by several authors (Yang *et al.*, 2013 ▸; Benvenuto *et al.*, 2016 ▸; Benvenuto, 2017 ▸; Bakry *et al.*, 2019 ▸). Although the convergence of the iterative EM algorithm is ensured since the algorithm is guaranteed to increase the likelihood with each iteration, a stable solution cannot be obtained because of its ill-posed nature and because an additional stabilization mechanism is required.

Several stabilization methods have been suggested for the EM algorithm. One of them is to add an additional smoothing operator into the iteration sequence. In *SASfit*, the smoothing operation suggested by Eggermont (1999 ▸), Eggermont & LaRiccia (1995 ▸) and Byrne & Eggermont (2011 ▸) has been implemented. For more details, refer to equations (36)–(55) in the supporting information.

As the EM algorithm is an iterative scheme, it can be extended by an additional entropy cost function, as shown by Richardson (1972 ▸) and Lucy (1974 ▸). In the work of Lucy (1994 ▸), two variants for introducing the maximum entropy cost function into the iteration algorithm are described: either using a known fixed prior or assuming an adaptive prior for the solution vector. These are the two other strategies for the EM iteration scheme made available in *SASfit*, and are detailed in the supporting information.

In all cases, the stabilization term or cost function is scaled by a weighting factor. To find the weighting factor, the same approaches have been used as in Tikhonov regularization by L-curve analysis (Hansen, 1998 ▸, 2001 ▸; Gazzola *et al.*, 2018 ▸).

In *SASfit*, the algorithms for determining the size distribution can be accessed via the menu bar of the main window under ‘Calc’ → ‘integral parameters’. This opens the ‘integral structural parameters’ window, which provides a selection box at the top where the retrieval algorithms are available for selection. The button ‘calculate N(R) using’ on the left side starts the algorithm using the input data from the main window.

**Figure 5:**
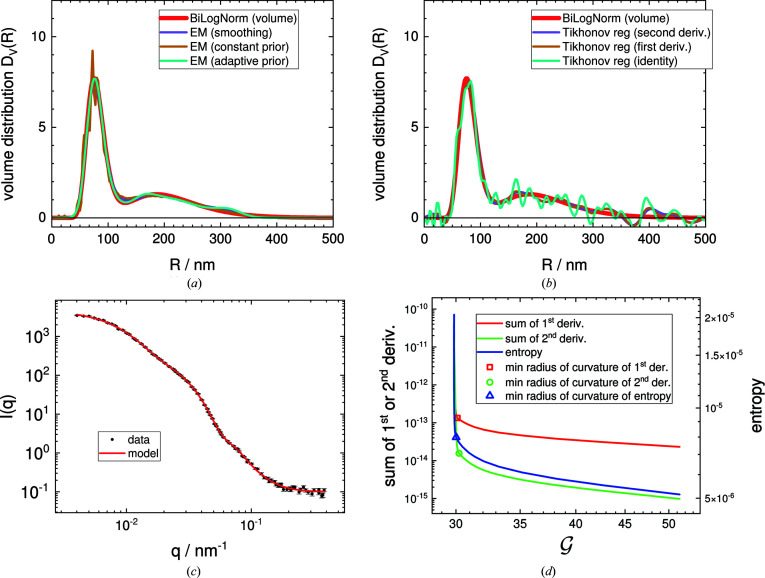
(*a*) Volume distributions *D*_V_(*R*) obtained by the different smoothing options in the EM iteration scheme. (*b*) Volume distributions *D*_V_(*R*) obtained by Tikhonov regularization. (*c*) Simulated experimental SAS data with noise and corresponding model scattering function. (*d*) The corner of the L-curve determines the optimum weighting factor for the cost function, which was chosen to be the entropy of the solution vector (blue) or the sum of its first (red) or second derivative (green), over the 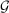 -test for the goodness of fit, which progresses from right to left with each iteration.

In Fig. 5 ▸, the different size-distribution retrieval algorithms are applied to a simulated scattering curve of a bimodal distribution with Gaussian noise added which was introduced for benchmarking by Jemian (2013 ▸) (file data/test.sas).

## 6. Structure factors

### 6.1. Ordered materials

**Figure 6:**
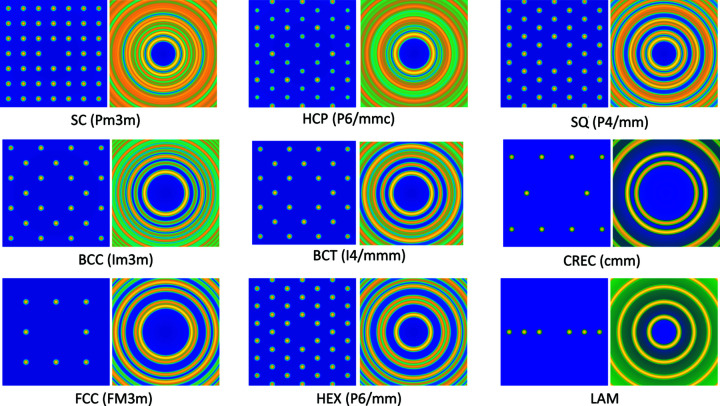
Structure factors of ordered mesoscopic structures, both for oriented crystals and their powder average. Structure factors of 3D crystals occurring in soft matter (SC, BCC, FCC, HCP, BCT – simple cubic structure, body-centred cubic, face-centred cubic, hexagonal close-packed, body-centered tetragonal), as well as 2D ordering (HEX, SQ, CREC) and 1D ordering (LAM), are supplied.

To describe the influence of the spatial arrangement of scatterers on the SAS signal, the structure factors need to be taken into account. However, an analytical description of the structure factor is available for only a limited number of models. For ordered nano- and meso-scaled materials, the scatterers show diffraction spots on the detector. For those systems, most of the structure factors from the software package *Scatter* (Förster *et al.*, 2010 ▸, 2005 ▸, 2011 ▸) are made available in *SASfit* for both aligned ordered structures and their powder averages. These models can be found in the ‘structure factor’ tab of the fit or simulation window under ‘by plugins’ → ‘ordered obj.’, where the ‘iso’ variants compute the structure factor for orientational averages and the ‘aniso’ variants compute for aligned ordered structures. A few examples are shown in Fig. 6 ▸.

### 6.2. Ornstein–Zernike solver

In the case of a simple liquid, in the framework of the OZ equations the structure factor can be calculated numerically on the basis of the known pair interaction potential between two particles and a choice of a closure relation. Therefore, *SASfit* now includes an OZ solver to calculate structure factors for spherically symmetric pair interaction potentials of scatterers. At the moment, only the monodisperse case is implemented. To account for polydispersity, approximations like the decoupling approach (Kotlarchyk & Chen, 1983 ▸), the local monodisperse approach (Pedersen, 1994 ▸) and a simple partial structure-factor model, as well as a scaling approximation of a partial structure factor (Gazzillo *et al.*, 1999 ▸), are implemented.

**Figure 7:**
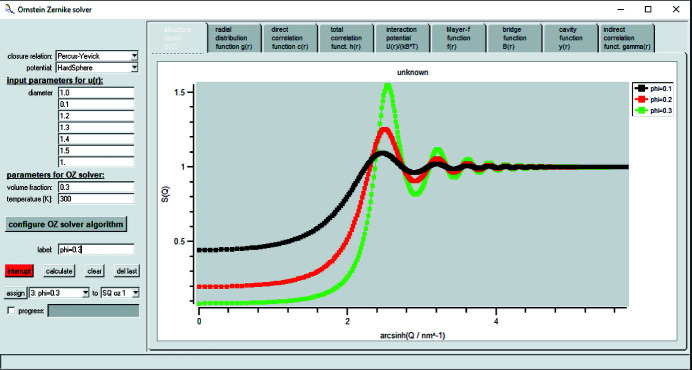
GUI for the OZ solver. The pair interaction potential together with a closure relation can be freely chosen by the user, as can the volume fraction of the particles. The internal algorithm can be configured as well, and the resulting structure factor is supplied by a special plug-in: *SQ calculated by OZ solver*. With this, however, only the radius can be fitted at the moment and all other parameters of the structure factor are fixed.

The GUI for the OZ solver is shown in Fig. 7 ▸. It allows one to choose a closure relation out of a set of 19 different closure relations and combine it with a pair interaction potential, ranging from sticky hard spheres to the soft sphere potential, several types of depletion potentials, the Yukawa potential, a piecewise constant potential, and Derjaguin–Landau–Verwey–Overbeek (DLVO), star-like or Lennard–Jones potentials, to name a few. The list of closure relations and potentials is extended continuously. Depending on whether the potential is short ranged or long ranged, the step size and total number of steps in real space need to be adapted.

We have compared the results of the numerical OZ solver in *SASfit* with known analytical solutions, which are also available in this software package. For a two-Yukawa potential with a hard core, Liu *et al.* (2005 ▸) have found an analytical solution using a closure for the mean spherical approximation (MSA). The potential reads as 

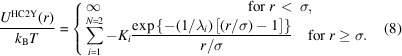

**Figure 8:**
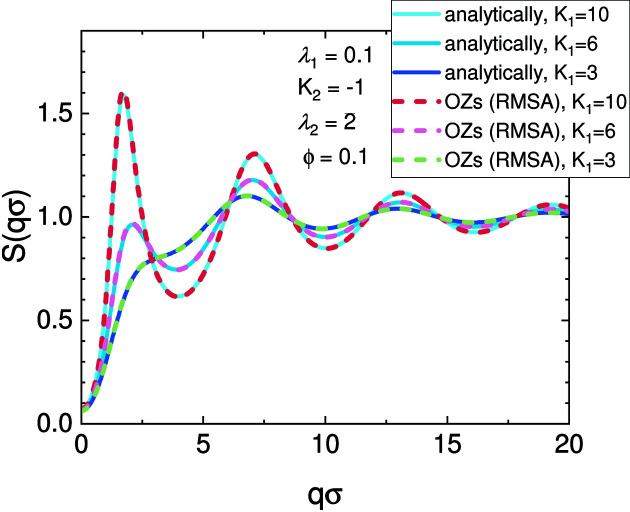
The numerical solutions of the OZ equations have been determined on a grid with 2^14^ = 16 364 points and a width of Δ*r* = σ/500, where σ is the hard core diameter. The parameters of the potentials are chosen so that they reproduce the cluster peak as discussed by Liu *et al.* (2005 ▸), caused by a long-range attractive interaction potential contribution. The solutions of the numerical OZ solver (dashed lines) overlay with the analytical solutions (solid lines). Therefore, the numerical OZ solver provides equivalent results to the analytical solutions.

In Fig. 8 ▸, the structure factor of the analytically solved two-Yukawa potential is compared with the numerical solution of the OZ equations using the same MSA closure. The differences between the two solutions are visually indistinguishable.

**Figure 9:**
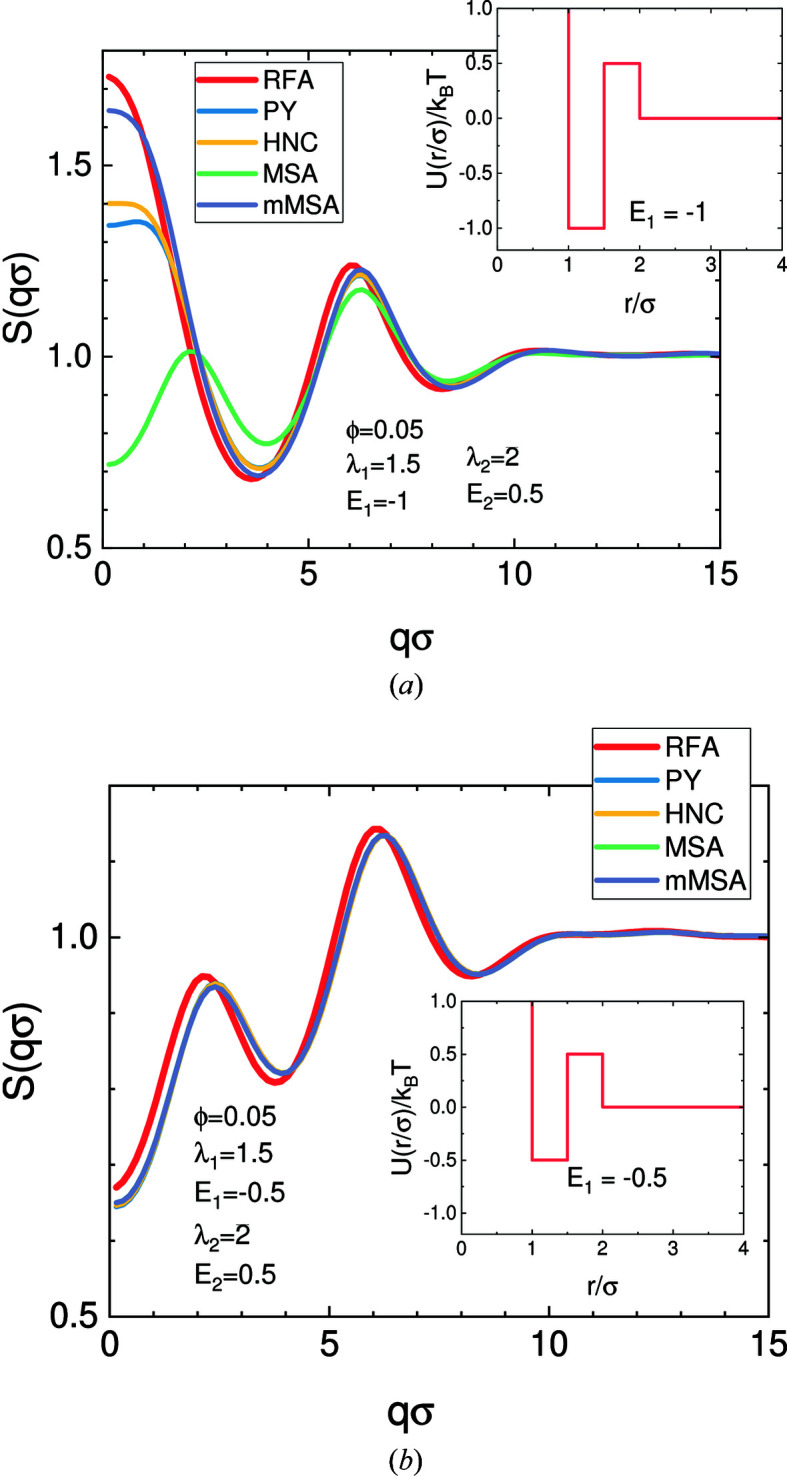
Structure factors of two different piecewise constant potentials shown in the insets are solved by the analytical solution of the RFA as well as by using different closures solved numerically with the OZ equations, namely Percus–Yevick (PY) (Percus & Yevick, 1958 ▸), hypernetted chain (HNC), MSA (Hansen & McDonald, 2013 ▸) and modified MSA (mMSA) (Gazzillo & Giacometti, 2003 ▸, 2004 ▸). For extreme values of the attractive well, such as those shown in (*a*), the closures might not be valid anymore and have to be verified against MD simulations; for more details, see the main text.

The closure recommended for a certain interaction potential is normally verified by MD simulations. Therefore, literature needs to be consulted to find the most appropriate combination of a potential and a closure. In the case of a piecewise linear potential, Santos *et al.* (2012 ▸, 2013 ▸) have found a quasi-analytical solution without introducing any closure relation, namely the ‘rational functional approximation’ (RFA). In Fig. 9 ▸ the RFA solution is compared with the numerical solution of the OZ solver using different closure relations. In the first case of a strong attractive well of *E*_1_ = −*k*_B_ *T* followed by a repulsive shoulder of *E*_2_ = *k*_B_ *T*/2, all closures show a significant difference from each other as well as a clear difference from the RFA approximation. To decide which closure is more appropriate for this potential, MD simulations would be needed. In the second example of Fig. 9 ▸, a less attractive well of *E*_1_ = −*k*_B_ *T*/2 has been chosen, and all closures as well as the RFA approximation yield very similar features. More details about solving the OZ equations can be found in the literature (Caccamo, 1996 ▸; Bomont, 2008 ▸; Hansen & McDonald, 2013 ▸; Santos, 2016 ▸; and references therein). Suggestions about combinations of potentials and the closures are also given there.

## 7. Conclusions and outlook

We have highlighted selected additions and updates to the *SASfit* program that may be most useful to interested users since the last publication concerning this software package. The technical foundation of the program is extended continuously with state-of-the-art algorithms according to the needs and challenges faced when new models and efficient problem-solving strategies are implemented. The plug-in system is one part of that foundation and it will be more extensively used in the future. New models are implemented as plug-ins by default, while older model functions from the early beginnings of the program will also be converted to plug-in functions. Recent changes to the plug-in system allow third parties to make use of the extensive library of models more easily and, moreover, they lay the groundwork to allow for interface packages to other programming languages. As an example, users can create Python-compatible modules from *SASfit* model plug-ins. For this endeavour, the current continuous integration setup is a suitable starting point. It builds software packages for all supported platforms on the fly and allows users to test code changes within hours instead of waiting for the next release. Prospectively, it will be extended to build further binary packages, for other programming languages, for example, and include more testing of basic program functions. Also, generating more of the available documentation automatically, such as the manual and the developer documentation, is planned.

In addition to new form and structure factors, some of which were presented here, a challenge for the future is the combination of regularization techniques with fits of multiple data sets from the same sample under different conditions. This scenario, also referred to as ‘global fitting’, aims to take advantage of the expanded information content available for fitting a form factor of the scatterers and employing regularization to determine a size distribution valid for all measurements considered. While this would not be possible with a single measurement, the additional information provided by multiple measurements could be sufficient to derive shape and size. The applicability of this scheme will only be proven by actual implementation.

We have presented the OZ solver as a versatile tool for numerically determining particle interactions and the resulting structure factor when no analytical solution is available, with the goal to include its effects in the SAS intensity model used for fitting with *SASfit*. At the moment it is a separate part of the program with little integration into the main curve-fitting workflow. Changes to achieve a more flexible integration of the numerically determined structure factors within the model setup are likely in future.

## 8. Related literature

The following additional references are cited in the supporting information for this article: Anderson (1965 ▸), Arslan *et al.* (2009 ▸), Barr (1981 ▸), Biggs (1998 ▸), Biggs & Andrews (1995 ▸, 1997 ▸), Blanc & Schlick (1996 ▸), Chae *et al.* (2019 ▸), Donatelli & Reichel (2014 ▸), Fougerolle *et al.*, 2005*a* ▸,*b* ▸, 2006 ▸, 2007 ▸), Freund (1993 ▸), Frigo & Johnson (1997 ▸), Gielis (2003 ▸), Hansen & O’Leary (1993 ▸), Hansen & Müller (1996 ▸), Henderson & Varadhan (2019 ▸), Homeier *et al.* (1995 ▸), Horne (1985 ▸), Jiang *et al.* (2017 ▸), Keelin *et al.* (2019 ▸), Kelley (2003 ▸), Kullback & Leibler (1951 ▸), Lewitt & Muehllehner (1986 ▸), Likos (2001 ▸), Niederreiter (1992 ▸), Saad (1993 ▸), Saad & Schultz (1986 ▸), Steenstrup & Hansen (1994 ▸), Svergun *et al.* (1988 ▸), Toth & Kelley (2015 ▸), van der Vorst (1992 ▸), Walker & Ni (2011 ▸) and Wang & Miller (2014 ▸). 

## Supplementary Material

Supporting information on SASfit updates. DOI: 10.1107/S1600576722009037/yr5094sup1.pdf
