# Supplementary material for: Updates in SASfit for fitting analytical expressions and numerical models to small-angle scattering patterns
Source: J Appl Crystallogr. 2022 Nov 21;55(Pt 6):1677–88. doi: 10.1107/S1600576722009037 (PMC9721323; doi:10.1107/S1600576722009037)
Supplement: Supplementary file 1 [file j-55-01677-sup1.pdf]

JOURNAL OF APPLIED CRYSTALLOGRAPHY

Volume 55 (2022)

Supporting information for article:

Updates in *SASfit* for fitting analytical expressions and numerical models to small-angle scattering patterns

Joachim Kohlbrecher and Ingo Breßler

JOURNAL OF APPLIED CRYSTALLOGRAPHY

Volume 55 (2022)

Supporting information for article:

Updates in *SASfit* for fitting analytical expressions and numerical models to small-angle scattering patterns

Joachim Kohlbrecher and Ingo Breßler

Supporting Information on SASfit updates

Joachim Kohlbrecher[a] and Ingo Bressler [b]*

[a]*Laboratory for Neutron Scattering and Imaging, Paul Scherrer Institut, 5232 Villigen PSI Switzerland, and* [b]*BAM Federal Institute for Materials Research and Testing, 12205 Berlin, Germany. E-mail: ingo.bressler@bam.de*

1 Form factors **2**

1.1 OPO - oriented primitive objects 2

1.2 Randomly oriented objects 5

1.3 Super-shapes 5

2 Size distributions **9**

2.1 MetaLog distribution 9

2.2 Regularization 10

2.3 Expectation Maximization 12

3 Structure factors from solving Ornstein-Zernike equations **26**

4 Parameters of numerical algorithms **32**

1. Form factors

1.1. OPO - oriented primitive objects

For many different geometric objects such as sphere, toroid, cone, pyramid or tetrahedron, analytical form factors are made available in SASfit. For analyzing anisotropic scattering patterns from such geometries SASfit expects one-dimensional SAS profiles in radial direction at a certain angle or in azimuthal direction.

A subclass of form factors in SASfit are oriented primitive objects. At the beginning this group was intended for simple single phase objects like homogeneous cube, cylinder, ellipsoid and their affine deformed shapes. We than added some other objects like platonic solids and super quadrics and further extended it by cones, pyramids, etc. All of them can be either perfectly oriented in the beam or are fully randomly oriented. For the perfect oriented version three Euler angles need to be supplied. Also many input parameters are needed for describing the affine deformation. The most simple geometric objects are unit spheres, unit cube and unit cylinder, and their affine deformed shapes of ellipsoid, oblique parallelepiped and oblique cylinder for which their $\mathbf{Q}$-dependent form factors $F(\mathbf{Q})$ are given analytically.

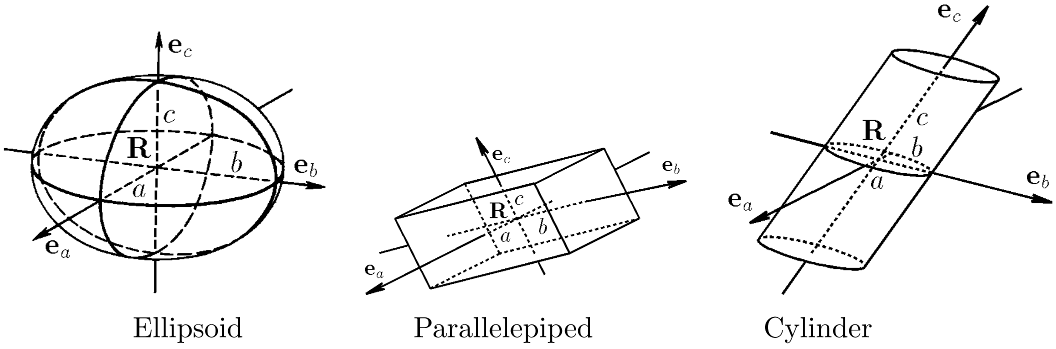

Fig. 1. $\mathbf{R}$: object center, a, b, c: half axes/lengths, $\mathbf{e}_a$, $\mathbf{e}_b$, $\mathbf{e}_c$: directions of the half axes

The scattering amplitude $F(\mathbf{Q})$ of an object is given by the Fourier transform of

the scattering length density distribution within the object

$$F(\mathbf{Q}) = \int_V \mathrm{d}\mathbf{r}\, \eta(\mathbf{r})\, e^{\imath \mathbf{Q}\mathbf{r}}, \qquad (1)$$

where $\eta(\mathbf{r})$ is the scattering length density distribution of the scattering object and V the sample volume. In the following it is assumed that the particles have a homogeneous scattering length density η_P, embedded in a matrix of also homogeneous scattering length density $\eta_M = \eta_P - \Delta\eta$. The position, orientation and size of the objects is uniquely defined by the center of scattering length density $\mathbf{R}$, the half axes a, b and c, as well as the direction of the half axes $\mathbf{e}_a$, $\mathbf{e}_b$ and $\mathbf{e}_c$. The base vectors $\mathbf{e}_i$ are normalised $|\mathbf{e}_i| = 1$ but do not need to be orthogonal to each other. For the scattering amplitude of an individual object the coordinate system can be changed after a translation by $-\mathbf{R}$ and a following change of basis $\underline{\mathbf{D}}$ from cartesian coordinates $O_{xyz} = \{\mathbf{e}_x, \mathbf{e}_y, \mathbf{e}_z\}$ into the coordinate system of the particle $O_{abc} = \{a\mathbf{e}_a, b\mathbf{e}_b, c\mathbf{e}_c\}$, so that $\mathbf{P}_{O_{abc}} = \underline{\mathbf{D}}\,\mathbf{P}_{O_{xyz}}$. With that relation in mind, the scattering amplitude in equation 1.1 can be written as:

$$F(\mathbf{Q}) = e^{-\imath \mathbf{Q}\mathbf{R}}\, \Delta\eta \int\limits_{V(\mathbf{0})} \mathrm{d}\mathbf{r}\, e^{\imath \mathbf{Q}(\underline{\mathbf{D}}^{-1}\underline{\mathbf{D}}\mathbf{r})} \qquad (2)$$

$$= \det(\underline{\mathbf{D}}^{-1})\, e^{-\imath \mathbf{Q}\mathbf{R}}\, \Delta\eta \int\limits_{\underline{\mathbf{D}}V(\mathbf{0})} \mathrm{d}\mathbf{r}\, e^{\imath \hat{\mathbf{Q}}\mathbf{r}} \qquad (3)$$

with

$$\hat{\mathbf{Q}} = (\underline{\mathbf{D}}^{-1})^T\, \mathbf{Q} = \begin{pmatrix} a\,\mathbf{e}_a\mathbf{Q} \\ b\,\mathbf{e}_b\mathbf{Q} \\ c\,\mathbf{e}_c\mathbf{Q} \end{pmatrix} \qquad (4)$$

$V(\mathbf{0})$ is the volume of the scattering object with a scattering length density center located at the origin of the coordinate system and $\underline{\mathbf{D}}V(\mathbf{0})$ is the volume of the unit object, i.e. unit sphere, unit cube or unit cylinder. The transformation matrix $\underline{\mathbf{D}}$ and

its inverse $\underline{\mathbf{D}}^{-1}$ are given by

$$\underline{\mathbf{D}}^{-1} = \begin{pmatrix} a\,e_{ax} & b\,e_{bx} & c\,e_{cx} \\ a\,e_{ay} & b\,e_{by} & c\,e_{cy} \\ a\,e_{az} & b\,e_{bz} & c\,e_{cz} \end{pmatrix} = (a\mathbf{e}_a, b\mathbf{e}_b, c\mathbf{e}_c) \tag{5}$$

$$\mathbf{Q}(\underline{\mathbf{D}}^{-1}\mathbf{r}) = ((\underline{\mathbf{D}}^{-1})^T\mathbf{Q})\mathbf{r}\,, \tag{6}$$

$$\begin{aligned} \det(\underline{\mathbf{D}}^{-1}) = abc\,(&e_{ax}\,e_{by}\,e_{cz} + e_{ay}\,e_{bz}\,e_{cx} + e_{az}\,e_{bx}\,e_{cy} \\ &- e_{az}\,e_{by}\,e_{cx} - e_{ay}\,e_{bx}\,e_{cz} - e_{ax}\,e_{bz}\,e_{cy}) \end{aligned} \tag{7}$$

$$\begin{aligned} \underline{\mathbf{D}} = (\underline{\mathbf{D}}^{-1})^{-1} &= \frac{1}{\det(\underline{\mathbf{D}}^{-1})}\,(\underline{\mathbf{D}}^{-1})^{\mathrm{adj}} \\ &= \frac{1}{\det(\underline{\mathbf{D}}^{-1})}\,(b\mathbf{e}_b \times c\mathbf{e}_c, -a\mathbf{e}_a \times c\mathbf{e}_c, a\mathbf{e}_a \times b\mathbf{e}_b) \end{aligned} \tag{8}$$

whereas $\det(\underline{\mathbf{D}})\,\det(\underline{\mathbf{D}}^{-1}) = \det(\underline{\mathbf{D}}\,\underline{\mathbf{D}}^{-1}) = \det(\mathbb{1}) = 1$

To reorient or rotate the objects the direction of the base vectors $\mathbf{e}_a$, $\mathbf{e}_b$, $\mathbf{e}_c$ needs to be adjusted which is done easily by introducing a rotation matrix $\underline{\mathbf{R}}_{\alpha\beta\gamma}$. The rotation matrix can be defined via Euler angles α, β and γ. Its definition depends on the convention used. There exist twelve possible sequences of rotation axes, divided in two groups: proper Euler angles (z-x-z, x-y-x, y-z-y, z-y-z, x-z-x, y-x-y) and Tait–Bryan angles (x-y-z, y-z-x, z-x-y, x-z-y, z-y-x, y-x-z). The difference between the two conventions is that Tait–Bryan angles represent rotations about three distinct axes, while proper Euler angles use the same axis for both the first and third elemental rotations. SASfit allows internally to use any of the 12 conventions. However, as default convention the yaw-pitch-roll is used (also named gier-nick-roll, east-north-up, north-East-Down or z-y-x is possible), so that

$$\hat{\mathbf{Q}} = \begin{pmatrix} a\,\underline{\mathbf{R}}_{\alpha\beta\gamma}\mathbf{e}_a\mathbf{Q} \\ b\,\underline{\mathbf{R}}_{\alpha\beta\gamma}\mathbf{e}_b\mathbf{Q} \\ c\,\underline{\mathbf{R}}_{\alpha\beta\gamma}\mathbf{e}_c\mathbf{Q} \end{pmatrix} \tag{9}$$

1.2. Randomly oriented objects

Next to the form factor of an oriented primitive object also the form factor of a fully randomly oriented version is made available. The orientational average is done by integrating over the full Ewald's sphere, which is a double integration. For this orientational average SASfit provides optimized integration routines which can be configured in the GUI shown in figure 1 of the article. Next to the multidimensional adaptive integration routines `pcubature` and `hcubature` (Johnson, 2020) also some other routines are supplied. However, these routines are nonadaptive without an error estimate of the integral but optimised for integrations over a sphere surface. Nevertheless, these routines often need significant less function evaluations than conventional multidimensional integration routines. The implemented methods are 2D-GaussLegendre integration, Lebedev (Lebedev, 1975; Lebedev, 1976; Lebedev, 1977) and Fibonacci (Niederreiter, 1992; Marques *et al.*, 2013) quadrature algorithm with a fixed and configurable number of function evaluations and without error estimation of the integral.

1.3. Super-shapes

The most flexible geometric objects made available in SASfit are so called super-shapes (Gielis, 2003; Arslan *et al.*, 2009; Fougerolle *et al.*, 2007) and rational super-shapes (Blanc & Schlick, 1996; Fougerolle *et al.*, 2007) which are a further generalisation of super-quadrics (Barr, 1981) widely used in computer graphics due to the property that their parametrisation allows a huge variety of shapes. These objects have both a parametric and an implicit representation. The parametric representation in two dimensions reads for super-shapes as (Gielis, 2003)

$$r(\theta,\alpha,m,n_1,n_2,n_3,a,b) = \left(\left|\frac{\cos\left(\frac{m(\theta-\alpha)}{4}\right)}{a}\right|^{n_2} + \left|\frac{\sin\left(\frac{m(\theta-\alpha)}{4}\right)}{b}\right|^{n_3}\right)^{-\frac{1}{n_1}} \tag{10}$$

and for rational super-shapes (Blanc & Schlick, 1996; Fougerolle *et al.*, 2007) as

$$r(\theta,\alpha,m,n_1,n_2,n_3,a,b) = 2^{\frac{-1}{n_1}}\left(\frac{1}{W(\theta-\alpha)n_1}+1-\frac{1}{n_1}\right) \tag{11}$$

with

$$W(\theta) = \frac{U(\theta)}{n_2+(1-n_2)U(\theta)} + \frac{V(\theta)}{n_3+(1-n_3)V(\theta)} \tag{12}$$

$$U(\theta) = \left|\frac{\cos\left(\frac{m\theta}{4}\right)}{a}\right| \quad \text{and} \quad V(\theta) = \left|\frac{\sin\left(\frac{m\theta}{4}\right)}{b}\right| \tag{13}$$

The generalization to extend the 2D parametric representation into 3D is done by a spherical product so that the parametrisation of a surface point $\mathbf{S}$ or within the volume $\mathbf{V}$ read as

$$\mathbf{S} = \begin{pmatrix} x(a_1,\theta,\phi) \\ y(a_2,\theta,\phi) \\ z(a_3,\theta,\phi) \end{pmatrix} = \begin{pmatrix} a_1\, r_1(\theta)\cos(\theta)\, r_2(\phi)\cos(\phi) \\ a_2\, r_1(\theta)\sin(\theta)\, r_2(\phi)\cos(\phi) \\ a_3\, r_2(\phi)\sin(\phi) \end{pmatrix} \tag{14}$$

$$\mathbf{V} = \tau\mathbf{S} \tag{15}$$

with

$$r_1(\theta) = r(\theta,\alpha,m,n_1,n_2,n_3,a,b) \tag{16}$$

$$r_2(\phi) = r(\phi,\beta,M,N_1,N_2,N_3,A,B) \tag{17}$$

where $\tau \in [0,1]$, $\theta \in [-\pi,\pi]$ and $\phi \in \left[-\frac{\pi}{2},\frac{\pi}{2}\right]$. For integer values of the degree of symmetries (Fougerolle *et al.*, 2005*a*; Fougerolle *et al.*, 2005*b*; Fougerolle *et al.*, 2006; Fougerolle *et al.*, 2007) have given 2 implicit representations for the super-shapes.

$$1 \geq \frac{x^2+y^2+z^2r_1^2(\theta)}{r_1^2(\theta)r_2^2(\phi)} \tag{18}$$

$$1 \geq 1-\frac{1}{r_2(\phi)}\sqrt{\frac{x^2+y^2+z^2}{\cos^2(\phi)\left(r_1^2(\theta)-1\right)+1}} \tag{19}$$

The angles θ and ϕ are defined by

$$\begin{cases} \theta &= \arctan\left(\frac{y}{x}\right) \\ \phi &= \arctan\left(\frac{zr_1(\theta)\sin(\theta)}{y}\right) \\ &= \arctan\left(\frac{zr_1(\theta)\cos(\theta)}{x}\right) \end{cases} \tag{20}$$

The Jacobi determinant for both the super-shape as well as rational super-shape substitution is

$$\det \underline{\mathbf{J}} = \det \begin{pmatrix} \frac{\partial x(\tau,\theta,\phi)}{\partial \tau} & \frac{\partial y(\tau,\theta,\phi)}{\partial \tau} & \frac{\partial z(\tau,\theta,\phi)}{\partial \tau} \\ \frac{\partial x(\tau,\theta,\phi)}{\partial \theta} & \frac{\partial y(\tau,\theta,\phi)}{\partial \theta} & \frac{\partial z(\tau,\theta,\phi)}{\partial \theta} \\ \frac{\partial x(\tau,\theta,\phi)}{\partial \phi} & \frac{\partial y(\tau,\theta,\phi)}{\partial \phi} & \frac{\partial z(\tau,\theta,\phi)}{\partial \phi} \end{pmatrix} = \tau^2 \cos(\phi) r_1^2(\theta) r_2^3(\phi) \tag{21}$$

$$F_{\mathrm{SuperSh}}(\mathbf{Q}) = \det(\underline{\mathbf{D}}^{-1})\, e^{-\imath \mathbf{QR}}\, \Delta\eta \int_0^1 \mathrm{d}\tau \int_{-\pi}^{\pi} \mathrm{d}\theta \int_{-\pi/2}^{\pi/2} \mathrm{d}\phi \, \det(\underline{\mathbf{J}}) e^{\imath \hat{\mathbf{Q}}\mathbf{r}} \tag{22}$$

$$= \det(\underline{\mathbf{D}}^{-1})\, e^{-\imath \mathbf{QR}}\, \Delta\eta \int_{-\pi}^{\pi} \mathrm{d}\theta \int_{-\pi/2}^{\pi/2} \mathrm{d}\phi \, \cos(\phi) r_1^2(\theta) r_2^3(\phi) \tag{23}$$

$$\times \left(\frac{2\hat{q}_s \cos\hat{q}_s + (\hat{q}_s^2 - 2)\sin\hat{q}_s}{\hat{q}_s^3} + \imath \frac{(2 - \hat{q}_s^2)\cos\hat{q}_s - 2 + 2q_s \sin\hat{q}_s}{\hat{q}_s^3} \right)$$

$$\hat{q}_s = \hat{\mathbf{Q}}\mathbf{S} = \hat{Q}_x x(1,\theta,\phi) + \hat{Q}_y y(1,\theta,\phi) + \hat{Q}_z z(1,\theta,\phi) \tag{24}$$

One of the three integrations can always be performed analytically. In figure 2 a selection of super-shapes and their scattering patterns are shown. The simulated scattering curve of the first example in the first column of figure 2 and the required input parameters are shown in figure 3.

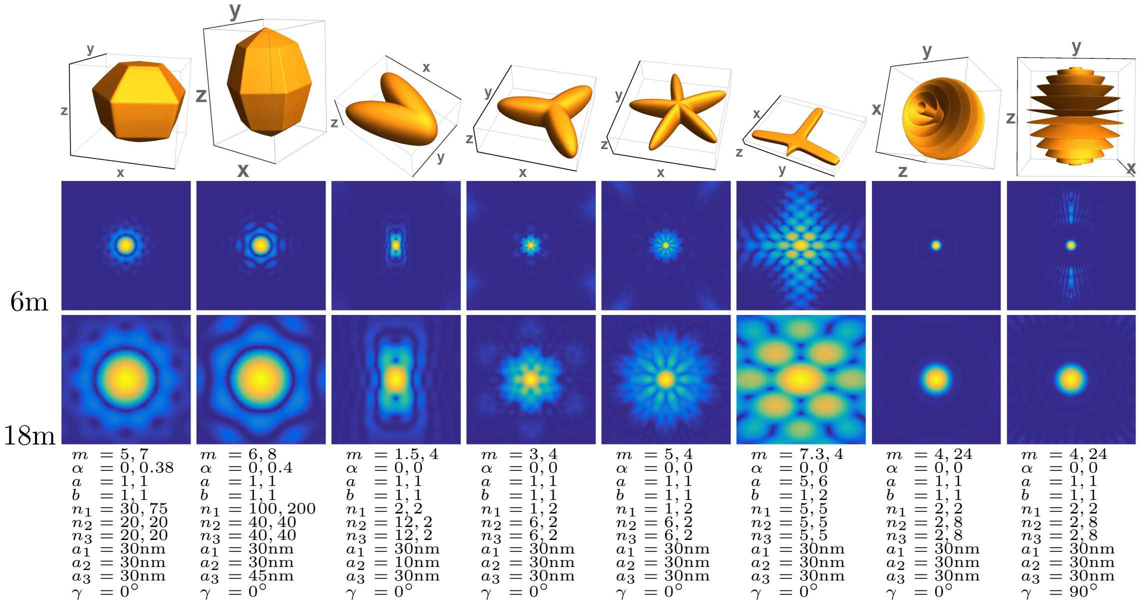

Fig. 2. Super-shapes: The scattering patterns are calculated for detector distances of 6 m and 18 m at a wavelength of 0.6 nm. The detector size is assumed to be $96 \times 96\text{cm}^2$. The values in the last row are the input values of eq. 10 for r_1 and r_2 (eqs. 16, 17). In the last example the incident beam direction is the x-directions instead of the z-direction.

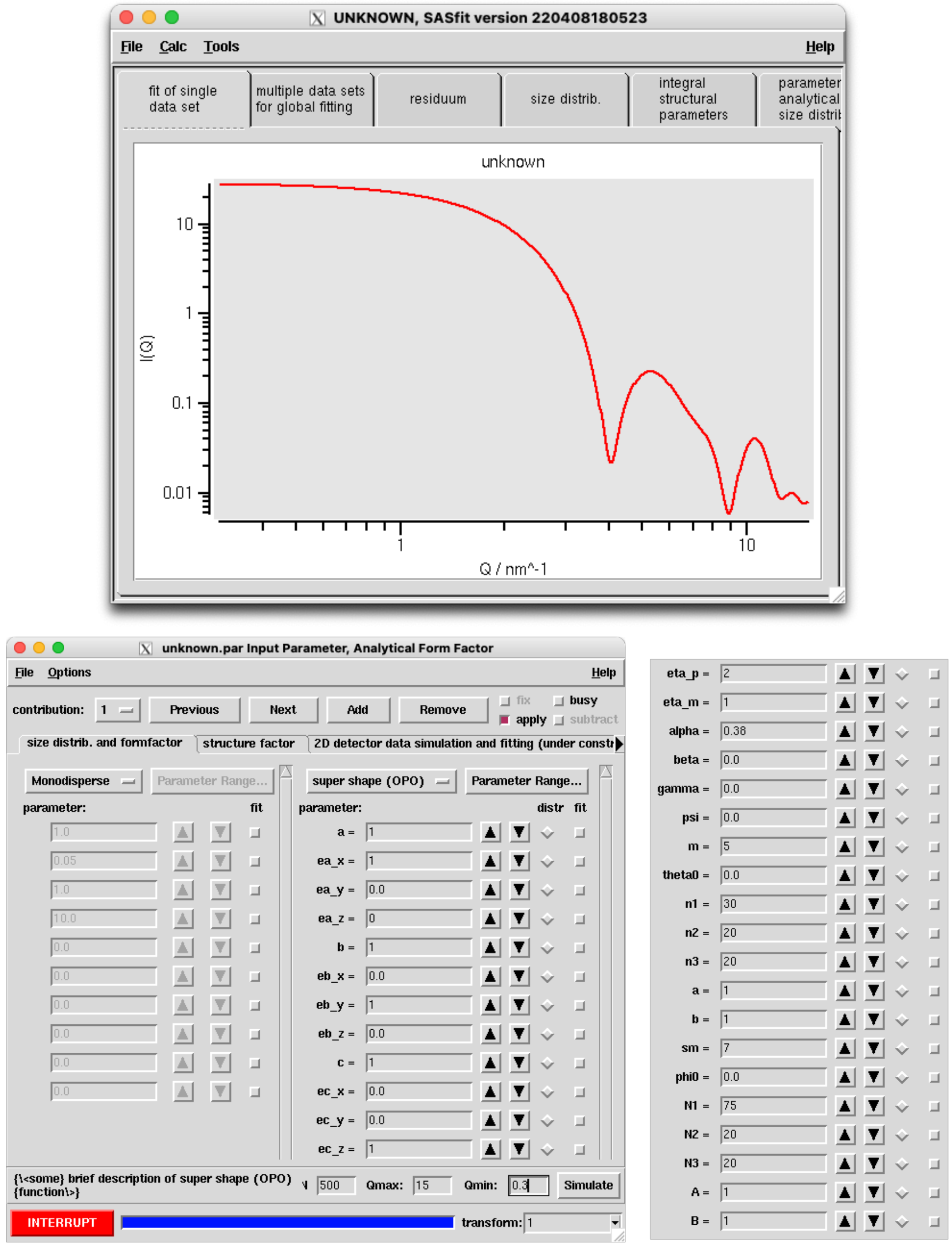

Fig. 3. Top: simulation plot of the first super-shape example given in figure 2 in the first column. Bottom: corresponding model parameters in SASfit for this super-shape example producing the plot above.

2. Size distributions

A standard task in analysing small-angle scattering data is the determination of the pair-distance distribution function or a size distribution of particles with a known form factor. Also desmearing of small-angle scattering data belongs to the same family of problems. The size distribution can be determined if the particle shape is known. For many particle shapes the scattering intensity or form factor can be calculated via an analytical expression. The scattering intensity is then described by integrating the squared form factor $F^2(Q,s)$ over a distribution function $N(s)$, where s describes the size of the scatterers (Pedersen, 1997):

$$I(Q) = \int_0^\infty N(s)\,|F(Q,s)|^2 \,\mathrm{d}s \tag{25}$$

To solve equation 25 often the size distribution is assumed to be an analytical distribution function such as LogNormal, Gaussian, Weibull or Schulz-Zimm with few input parameters. These parameters are then optimised by performing the numerical integration and applying a Levenberg-Marquardt strategy to solve a nonlinear least squares problem (Breßler *et al.*, 2015). This however assumes a priori knowledge about the distribution function.

2.1. MetaLog distribution

A distribution function designed to be very flexible is the metalog distribution. The quantile function of the metalog distribution $Q(y) = M_k(y)$ is defined as (Keelin & Powley, 2011; Keelin, 2016; Keelin *et al.*, 2019)

$$M_k(y) = \begin{cases} a_1 + a_2 \ln \frac{y}{1-y}, & k = 2 \\ a_1 + a_2 \ln \frac{y}{1-y} + a_3(y-\frac{1}{2}) \ln \frac{y}{1-y}, & k = 3 \\ a_1 + a_2 \ln \frac{y}{1-y} + a_3(y-\frac{1}{2}) \ln \frac{y}{1-y} + a_4(y-\frac{1}{2}), & k = 4 \\ M_{k-1}(y) + a_k(y-\frac{1}{2})^{\frac{k-1}{2}}, & \text{odd } k \geq 5 \\ M_{k-1}(y) + a_k(y-\frac{1}{2})^{\frac{k}{2}-1} \ln \frac{y}{1-y}, & \text{even } k \geq 6 \end{cases} \tag{26}$$

and its PDF $p(x) = m_k(y)$ with $x = M_k(y)$ reads as

$$m_k(y) = \begin{cases} \frac{y(1-y)}{a_2}, & k=2 \\ \left(\frac{a_2}{y(1-y)} + a_3\left(\frac{y-\frac{1}{2}}{y(1-y)} + \ln\frac{y}{1-y}\right)\right)^{-1}, & k=3 \\ \left(\frac{a_2}{y(1-y)} + a_3\left(\frac{y-\frac{1}{2}}{y(1-y)} + \ln\frac{y}{1-y}\right) + a_4\right)^{-1}, & k=4 \\ \left(\frac{1}{m_{k-1}(y)} + a_k\frac{k-1}{2}(y-\frac{1}{2})^{(k-3)/2}\right)^{-1}, & \text{odd } k \geq 5 \\ \left(\frac{1}{m_{k-1}(y)} + a_k\left(\frac{(y-\frac{1}{2})^{\frac{k}{2}-1}}{y(1-y)} + (\frac{k}{2}-1)(y-\frac{1}{2})^{(\frac{k}{2}-2)}\ln\frac{y}{1-y}\right)\right)^{-1}, & \text{even } k \geq 6 \end{cases} \quad (27)$$

The metalog PDF as defined above is an unbounded distribution. In case of describing a size distribution it needs to be bounded (Logit metalog) or at least semi-bounded (bounded below, Log metalog). This can be easily done by a transformation of $z(x) = \ln(x - b_l)$ for a distribution with a lower bound b_l or $z(x) = \ln\left(\frac{x-b_l}{b_u-x}\right)$ with a lower bound b_l and an upper bound b_u, where z is then metalog-distributed. The corresponding quantile functions and PDF read as

$$M_k^{\log}(y) = \begin{cases} b_l + e^{M_k(y)}, & 0<y<1 \\ b_l, & y=0 \end{cases} \quad (28)$$

$$m_k^{\log}(y) = \begin{cases} m_k(y)e^{-M_k(y)}, & 0<y<1 \\ 0, & y=0 \end{cases} \quad (29)$$

$$M_k^{\text{logit}}(y) = \begin{cases} \frac{b_l+b_u e^{M_k(y)}}{1+e^{M_k(y)}}, & 0<y<1 \\ b_l, & y=0 \\ b_u, & y=1 \end{cases} \quad (30)$$

$$m_k^{\text{logit}}(y) = \begin{cases} m_k(y)\frac{\left(1+e^{M_k(y)}\right)^2}{(b_u-b_l)e^{M_k(y)}}, & 0<y<1 \\ 0, & y=0 \\ 0, & y=1 \end{cases} \quad (31)$$

2.2. Regularization

Instead of using an analytical description of the size distribution it would be more desirable to use a discretization of the size distribution N_i at the positions s_i and determine all N_i without any further assumptions.

Mathematically, these problems are equivalent to finding a solution for a Fredholm integral of the first kind. These tasks are ill-posed problems by their nature and an

additional regularization technique has to be included in the analysis to obtain a stable solution (Tikhonov, 1943; Tikhonov *et al.*, 1995). For small-angle scattering this technique has been introduced by (Glatter, 1977) and later on taken over by (Svergun *et al.*, 1988) and (Hansen & Pedersen, 1991). To find a stable solution a penalty term in the minimization procedure had to be included which introduced the question how to properly weight this penalty term. An early discussion about this for small-angle scattering can be found in (Svergun, 1992). However, the proper weighting of the penalty has been treated also in other fields and a discussion can be found for example in (Hansen & O'Leary, 1993; Hansen, 2000). Fredholm integrals, like the one in eq. 25, can be written as linear matrix equations $\boldsymbol{A}\mathbf{x} = \mathbf{b}$ by discretization. Hereby, $\boldsymbol{A}$ is a $M \times N$ matrix, $\mathbf{b}$ is a vector of length M and $\mathbf{x}$ is a solution vector of length N. Their elements are given by

$$x_j = N(s_j) s_j^{\alpha} \tag{32}$$

$$b_i = I(Q_i) \tag{33}$$

$$A_{i,j} = \Delta s_j \, s_j^{-\alpha} \, |F(Q, s_j)|^2 \tag{34}$$

$s_j^{-\alpha} \| F(Q, s_j) \|^2$ are known functions. The terms s_j^{α} and $s_j^{-\alpha}$ have been introduced to obtain either a number size distribution ($\alpha = 0$), volume weighted size distribution ($\alpha = 3$), or an intensity weighted size distribution ($\alpha = 6$).

The solution vector $\mathbf{x}$ is then obtained by a least squares optimization. If the penalty term $\boldsymbol{L}$ can also be linearized the problem can be reduced to solving the linear least squares problem

$$\chi^2 = ||\mathbf{b} - \boldsymbol{A}\mathbf{x}||_w^2 + \lambda^2 ||\boldsymbol{L}\mathbf{x}||^2 \tag{35}$$

where $|| \dots ||_w^2$ denotes the weighted sum of squared residuals and λ the regularization parameter weighting the penalty term. Typically the linear least squares equation has to be solved for many λ and a criteria of finding the proper regularization parameter

has to be chosen (Hansen & O'Leary, 1993). For the linear penalty term typically the identity matrix or the first or second order derivative operator are taken (Donatelli & Reichel, 2014). Using the entropy with a positive prior as a penalty function the solution vector is also automatically constrained to positive values. Maximum Entropy methods have been used for small-angle scattering analysis, for example by (Hansen & Müller, 1996; Hansen & Pedersen, 1991). (Steenstrup & Hansen, 1994) have shown how to use Maximum Entropy methods without the positivity constraint.

2.3. Expectation Maximization

Expectation maximization (EM) algorithm is a procedure for designing iterative methods for likelihood maximization. This framework has been explained first by (Dempster *et al.*, 1977). Applied to Fredholm integrals the method is an iterative fixed point method for non-negative real valued functions (Vardi & Lee, 1993). The method described there is equivalent to the Lucy-Richardson method (Richardson, 1972; Lucy, 1974) which is well known and widely used in image analysis.

Recently, expectation maximization (EM) methods for inverting Fredholm integrals in small-angle scattering have been studied for obtaining size distribution information (Yang *et al.*, 2013; Benvenuto *et al.*, 2016; Benvenuto, 2017; Bakry *et al.*, 2019). For inverting scattering data to pair distance distribution functions the condition of non-negativity of the involved functions does not hold anymore as both the kernel of the Fredholm integral as well as the pair distance distribution function can take negative values. In (Chae *et al.*, 2018) it has been shown, how the EM algorithm can be reformulated for non-density functions, i.e. to functions which also can take negative

values. The EM iteration scheme or Lucy-Richardson inversion method reads as

$$x_j^{(k+1)} = x_j^{(k)} + \Delta x_j^{(k)} = \mathcal{O}_{\mathrm{EM}}\left[x_j^{(k)}\right] \tag{36}$$

$$\Delta x_j^{(k)} = x_j^{(k)}\left[\sum_{i=0}^{M-1} \frac{A_{ij}}{\left(\sum_{m=0}^{M-1} A_{mj}\right)} \frac{b_i}{\sum_{n=0}^{N-1} A_{in} x_n^{(k)}} - 1\right] \tag{37}$$

where $\mathcal{O}_{\mathrm{EM}}$ is the fixed point operator. It has been shown, that the convergence is assured since the algorithm is guaranteed to increase the likelihood at each iteration. On the other side the algorithm is known to converge quite slow.

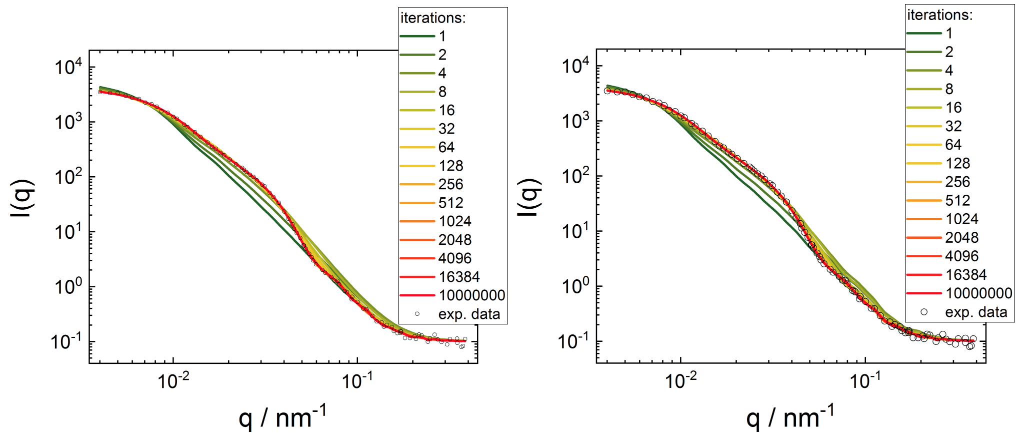

Fig. 4. Scattering curve evolution for increasing number of iterations for a constant seed value (left) and random seed values (right).

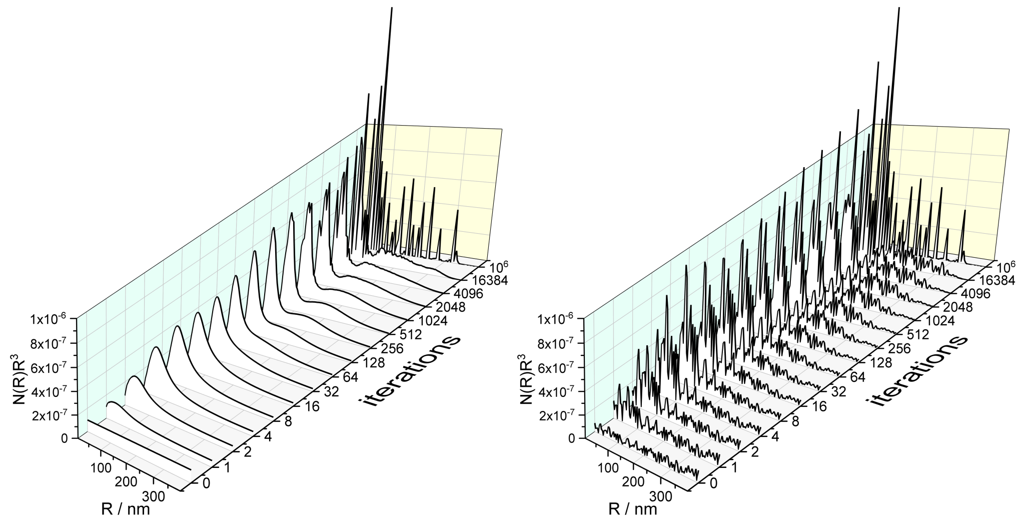

Fig. 5. Size distribution evolution for increasing number of iterations for a constant seed value (left) and random seed values (right).

In figure 4 and 5 the progress of the EM-fixed point algorithm as a function of the number of iteration steps is shown. The starting values of the size distribution are assumed to be a constant (left) and random (right). In both cases the EM-scheme converges to the same solution finally. However, assuming constant initial values the size distribution looks relatively smooth until the goodness-of-fit parameter approaches its final value in contrast to random seed values for the size distribution where the initial roughness is never smoothed out by the EM scheme. In figure 6 the goodness-of-fit parameters are shown as a function of iterations. The following three variants of goodness-of-fit definitions are used: the classical χ_r^2 of reduced weighted sums of squared residuals, the $\mathcal{G}$-test which is directly related to the Kullback-Leibler divergency (Kullback & Leibler, 1951) of two probability functions and the Pearson's χ_P^2-test, which is obtained by a second order Taylor expansion of the natural logarithm

around 1 of the $\mathcal{G}$-test.

$$\chi_r^2 = \sum_i^N \frac{1}{N} \left(\frac{I_{\text{th}}(q_i) - I_{\text{exp}}(q_i)}{\Delta I_{\text{exp}}(q_i)} \right)^2 \qquad \text{weighted sum of squared residuals} \tag{38}$$

$$\chi_P^2 = \sum_i \frac{(I_{\text{th}}(q_i) - I_{\text{exp}}(q_i))^2}{I_{\text{th}}(q_i)} \qquad \text{Pearson's } \chi^2\text{-test} \tag{39}$$

$$\mathcal{G} = 2 \sum_i \mathcal{I}_{\text{exp}}(q_i) \ln \left(\frac{\mathcal{I}_{\text{exp}}(q_i)}{\mathcal{I}_{\text{th}}(q_i)} \right) \qquad \mathcal{G}\text{-test} \tag{40}$$

with $\mathcal{I}_{\text{exp}}(q_i) = I_{\text{exp}}(q_i)/\sum_i I_{\text{exp}}(q_i)$ and $\mathcal{I}_{\text{th}}(q_i) = I_{\text{th}}(q_i)/\sum_i I_{\text{th}}(q_i)$. Furthermore, the roughness of the resulting size distribution is plotted which has been described by either the sum of the squared first or second derivatives of all radii, i.e. $\sum_i |\mathrm{d}N(R_i)/\mathrm{d}R_i|^2$ or $\sum_i |\mathrm{d}^2N(R_i)/\mathrm{d}R_i^2|^2$.

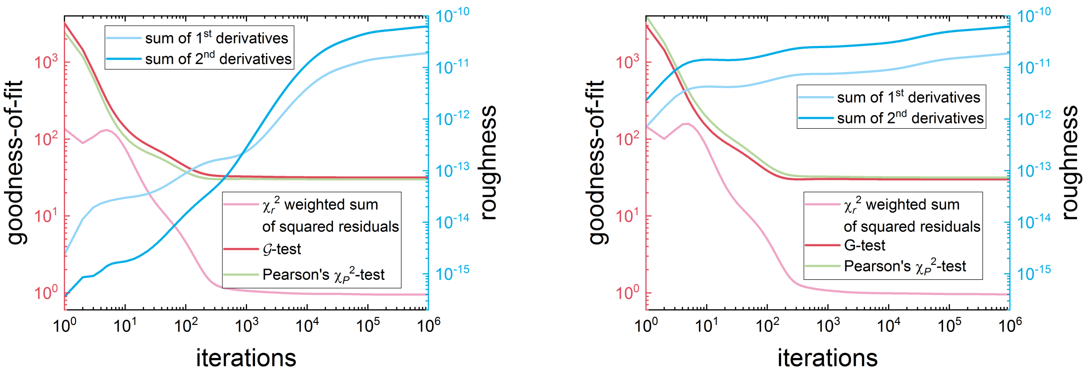

Fig. 6. Dependency of goodness-of-fit and roughness on number of iterations for a constant seed value (left) and random seed values (right).

The roughness parameter and the goodness-of-fit parameter are then combined in figure 7 on loglog-scale showing a similar behaviour as the L-curve in regularization techniques solving ill-posed problems. After a certain number of iterations the goodness-of-fit only improves infinitesimal but the roughness still continues to grow for a quite large number of iterations before it reaches the fixed point.

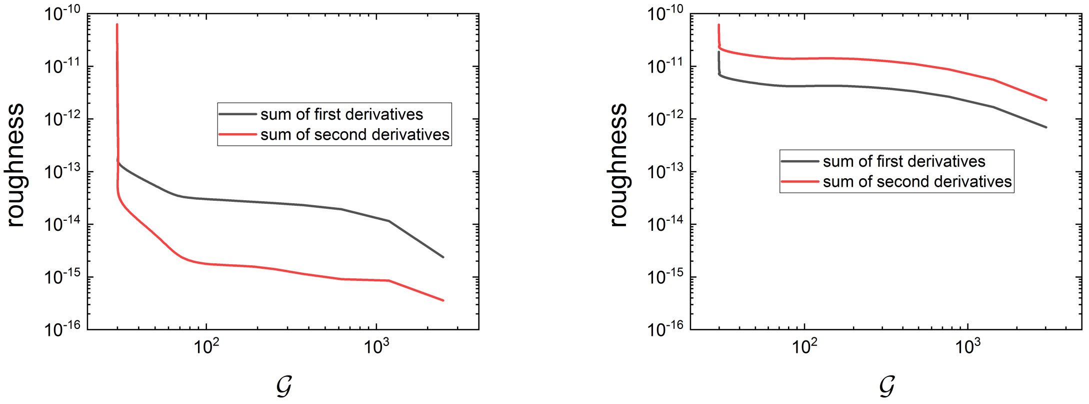

Fig. 7. L-curves showing the dependency of the roughness value over the $\mathcal{G}$-test goodness-of-fit value for a constant seed value (left) and random seed values (right). The goodness-of-fit value (x-axis) progresses from right to left (getting smaller) with each iteration of the fitting routine.

Even though the fixed point of the EM operator does not seem to depend on the initialization of the starting values, it will depend on the actual noise in the data, i.e. if the curve would be measured a second time with similar statistics, the two curves would differ within their noise level and the EM fixed point would then be slightly different which is caused by the ill-posed nature of the Fredholm integral. Two strategies for overcoming this problem have been tested. One strategy introduces a smoothing operator into the iteration loop and a second strategy introduces a classical regularization term (aka. penalty) in the EM scheme, which both then require a determination of the smoothing or regularization parameter.

Smoothing Operator In SASfit the smoothing operation suggested by (Eggermont, 1999; Eggermont & LaRiccia, 1995; Byrne & Eggermont, 2011) has been implemented. They suggest to add before or/and after each normal EM step (eq. 36) an additional

smoothing step so that the EM algorithm takes the form.

$$x_j^{(k+1/2)} = x_j^{(k)} + \Delta x_j^{(k)} \tag{41}$$

$$x_j^{(k+1)} = \sum_{i=0}^{M-1} S_{ij}^{(h)} x_j^{(k+1/2)} \tag{42}$$

with the smoothing operator

$$\boldsymbol{S}^{(h)} = \begin{pmatrix} 1-h & h & 0 & \cdots & 0 & 0 \\ h & 1-2h & h & \cdots & 0 & 0 \\ 0 & h & 1-2h & \cdots & 0 & 0 \\ \vdots & \vdots & \vdots & \ddots & \vdots & \vdots \\ 0 & 0 & 0 & \cdots & 1-2h & h \\ 0 & 0 & 0 & \cdots & h & 1-h \end{pmatrix} \tag{43}$$

whereby $0 < h \lessapprox 0.3$. In case of a double smoothing they suggest a nonlinear smoothing operator before each EM step so that a complete smoothed EM step reads as

$$x_j^{(k+1/3)} = \exp\left(\sum_{i=0}^{M-1} S_{ij}^{(h)} \log\left(x_j^{(k)}\right)\right) \tag{44}$$

$$x_j^{(k+2/3)} = x_j^{(k+1/3)} + \Delta x_j^{(k+1/3)} \tag{45}$$

$$x_j^{(k+1)} = \sum_{i=0}^{M-1} S_{ij}^{(h)} x_j^{(k+2/3)} \tag{46}$$

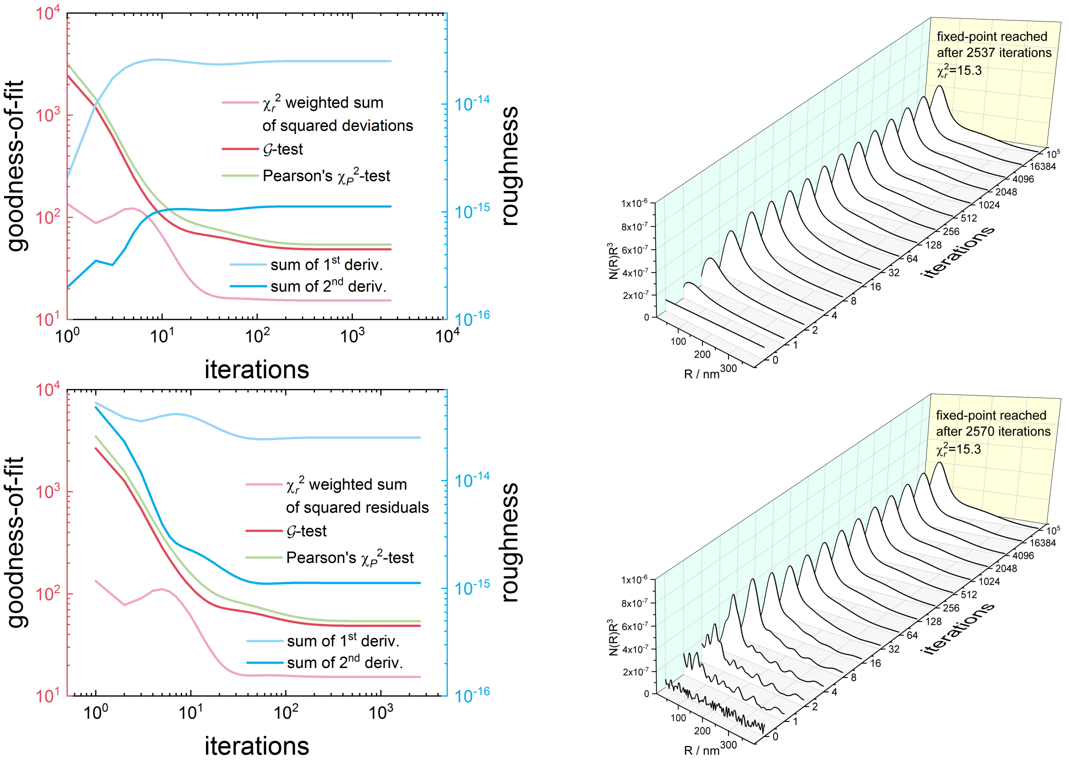

Fig. 8. Roughness and goodness-of-fit parameters as well as the resulting volume distribution $N(R)R^3$ as a function of iterations for a constant seed value (top) and random seed values (bottom) for a large smoothing parameter $h = 0.3$ leading to $\chi_r^2 = 15.3$.

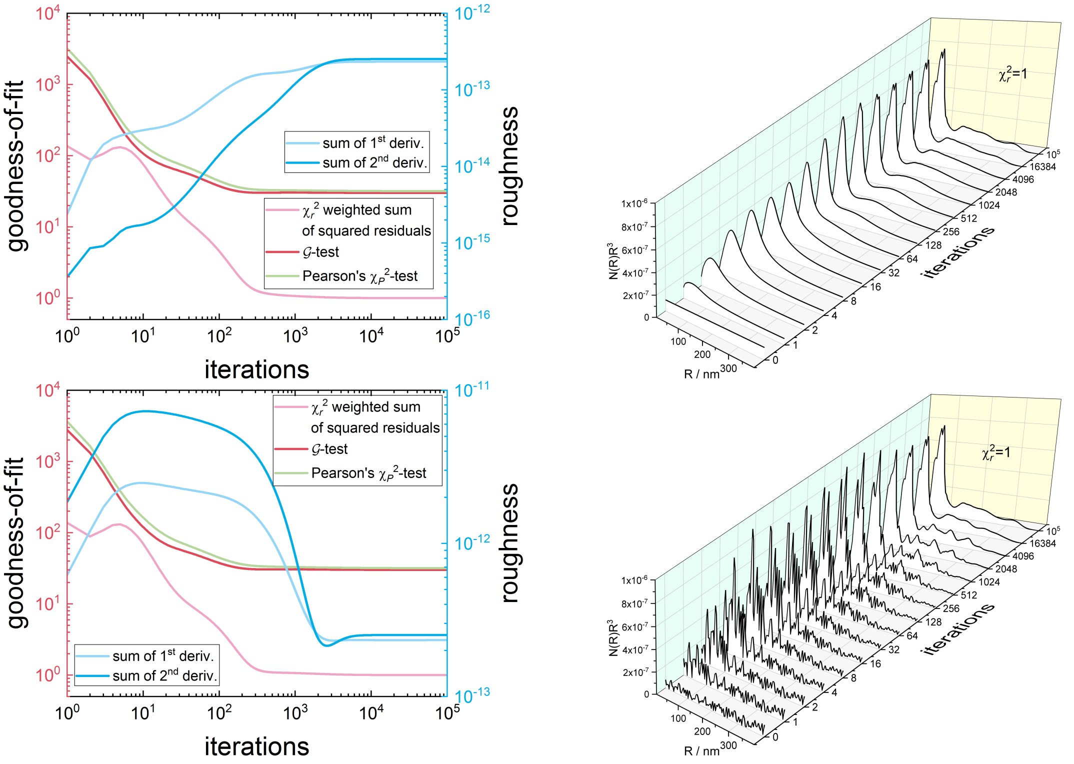

Fig. 9. Roughness and goodness-of-fit parameters as well as the resulting volume distribution $N(R)R^3$ as a function of iterations for a constant seed value (top) and random seed values (bottom) for an optimum smoothing parameter $h = 1.87 \times 10^{-4}$ leading to $\chi_r^2 = 1$.

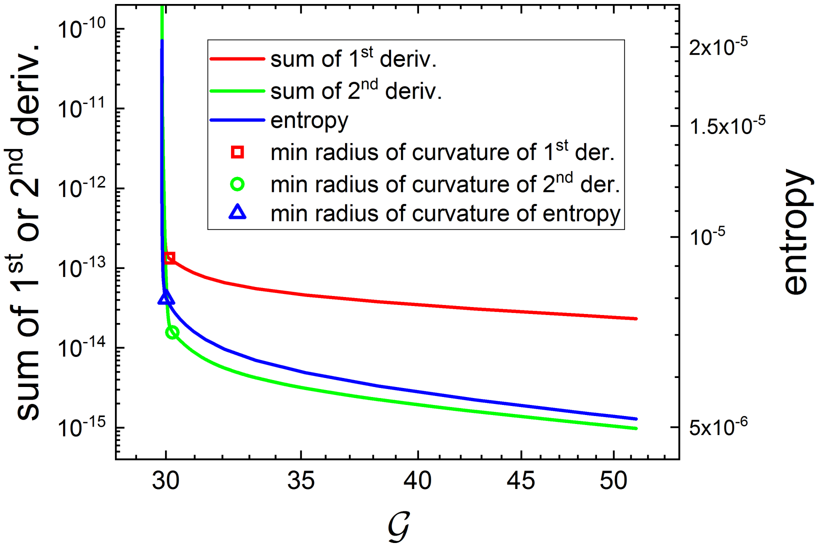

Fig. 10. Optimum smoothing parameter h given by the corner of the L-curve.

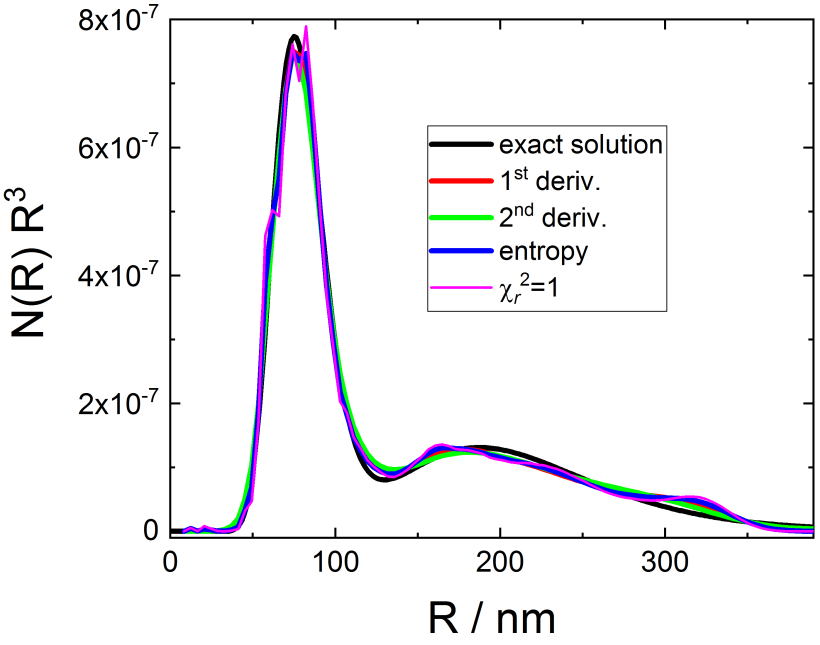

Fig. 11. The smoothing parameters have been obtained by the corners of the L-curves in fig. 10 and the solution of the EM iteration scheme with double smoothing is compared with the exact solution.

The proper choice of the parameter h arises and its selection might follow similar strategies than for the regularization parameter in ill-posed problems. In contrast to regularization methods, in this iteration scheme there is not directly a penalty term involved such as measuring the roughness of the solution by summing up the first or second derivatives of the solution vector or by measuring the entropy of the solution vector as in maximum entropy methods. However, these quantities can easily be calculated from the solution vector. Figure 7 shows the logarithm of a roughness term, namely the sum of 1st and 2nd derivatives of the resulting size distribution and the entropy against the goodness-of-fit parameter from eq. 40, namely the "$\mathcal{G}$-test" parameter. The optimum smoothing parameter has been defined via the corner of the L-curve defined by $(\log(\mathcal{G}), \log(||\hat{\mathbf{L}}\mathbf{x}||^2))$, with $\hat{\mathbf{L}}$ being the first or second-order discrete derivative operator. for example given in (Donatelli & Reichel, 2014) and by $(\log(\mathcal{G}), \log(S))$ with S being the entropy defined in eq. 47. The corners were

determined numerically as they are defined by having the maximum curvature. They are marked in fig. 10 by a symbol. The whole analysis does not make use of the uncertainties of the measured intensities. If the error bars are trustable and the model for the form factor is correct the weighted sum of squared residuals should be $\chi_r^2 = 1$. This can also be used as a condition for determining the smoothing parameter h. In the case of the present analysed data both strategies yield similar results as can be seen in fig. 11.

Penalty function The Lucy-Richardson method (Richardson, 1972; Lucy, 1974) has been extended for an additional penalty function, namely the entropy (Lucy, 1994). The maximum entropy penalty can be introduced in the iteration algorithm by knowing either a fixed or assuming an adaptive prior for the solution vector. The entropy S is given by

$$S = \sum_{j=0}^{N-1} -x_j \ln\left(x_j/m_j\right) + x_j - m_j \tag{47}$$

with $\mathbf{m}$ being the prior estimate of $\mathbf{x}$. The update scheme in eq. 36 has to be extended as

$$x_j^{(k+1)} = x_j^{(k)} + \Delta x_j^{(k)} + \Delta s_j^{(k)} = \mathcal{O}_{\mathrm{EM,ME,const}}\left[x_j^{(k)}\right] \tag{48}$$

with

$$\Delta x_j^{(k)} = x_j^{(k)} \left[\sum_{i=0}^{M-1} \frac{A_{ij}}{\left(\sum_{m=0}^{M-1} A_{mj}\right)} \frac{b_i}{\sum_{n=0}^{N-1} A_{in} x_n^{(k)}} - 1 \right] \tag{49}$$

$$\Delta s_j^{(k)} = \lambda x_j^{(k)} \left(-\ln \frac{x_j}{m_j} + \frac{1}{\sum_{j=0}^{N-1} x_j^{(k)}} \sum_{j=0}^{N-1} x_j^{(k)} \ln \frac{x_j^{(k)}}{m_j} \right) \tag{50}$$

in case of a known prior. If a prior is not known a constant prior can be chosen. However, even if no model for the prior is known, one can try to construct one adaptively according to Horne (Horne, 1985) by applying for example a Gaussian point spread

function to the actual solution and using this as a prior for the next iteration step. In this case the update scheme changes to

$$x_j^{(k+1)} = x_j^{(k)} + \Delta x_j^{(k)} + \Delta s_j^{(k)} = \mathcal{O}_{\text{EM,ME,adaptive}}\left[x_j^{(k)}\right] \tag{51}$$

$$\Delta x_j^{(k)} = x_j^{(k)}\left[\sum_{i=0}^{M-1} \frac{A_{ij}}{\left(\sum_{m=0}^{M-1} A_{mj}\right)} \frac{b_i}{\sum_{n=0}^{N-1} A_{in} x_n^{(k)}} - 1\right] \tag{52}$$

$$\begin{aligned}\Delta s_j^{(k)} = \lambda x_j^{(k)} \Bigg(& \frac{1}{\sum_{j=0}^{N-1} x_j^{(k)}} \sum_{j=0}^{N-1} x_j^{(k)} \ln \frac{x_j^{(k)}}{m_j^{(k)}} \\ & - \ln \frac{x_j}{m_j^{(k)}} - 1 + \sum_{i=0}^{N-1} \Pi_{ij} \frac{x_i^{(k)}}{m_i^{(k)}} \Bigg)\end{aligned} \tag{53}$$

$$m_i^{(k)} = \sum_{j=0}^{N-1} \Pi_{ij} x_j^{(k)} \tag{54}$$

with a smoothing operator Π_{ij} for the prior:

$$\Pi_{ij} = \frac{1}{c} \exp\left(-\frac{(i-j)^2}{2\sigma^2}\right), \tag{55}$$

a normalization constant $c = \sum_j \Pi_{ij}$ and a σ^2 typically between $\frac{1}{2}$ and 2. For large values of σ^2 the results converge to those of a constant prior. If the values are too small the regularization is suppressed as the prior converges to the kth iteration step.

The optimal regularization parameter λ can be determined similar to the previous section by the corner of the L-curve. The L-curves for both a constant and adaptive prior are shown in fig. 12 using the same data shown in fig. 4. The Lagrange parameter optimum in the corner is marked by a symbol. The corresponding size distributions are shown in fig. 13 together with the exact solution and for a Lagrange parameter leading to $\chi_r^2 = 1$. The L-curves in fig. 12 show that the $\mathcal{G}$-test parameter for the constant prior varies over several orders of magnitudes whereas the adaptive prior, even with a Lagrange parameter far away from its optimal value leads to a reasonable good result. Therefore, the L-curve shows a much smaller range on the x-axis for the adaptive prior.

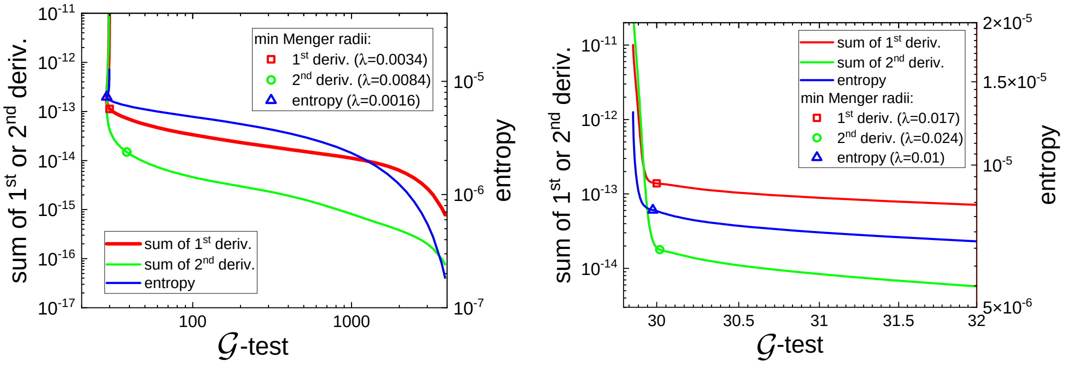

Fig. 12. Determine the optimal smoothing parameter λ by the corner of the L-curve which is given by the smallest radius of curvature (Menger radius). Left: constant prior, Right: adaptive prior

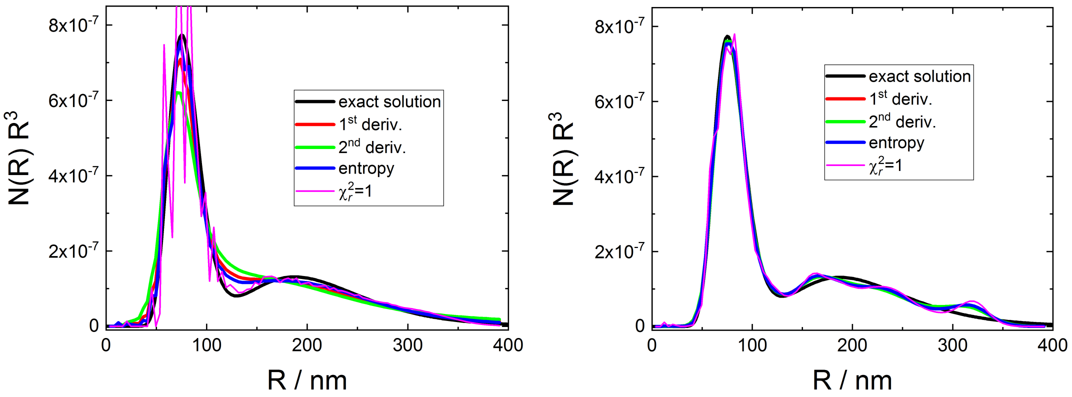

Fig. 13. The smoothing parameters that were obtained by the corners of the L-curves in fig. 12 and the solution of the EM iteration scheme using Maximum Entropy term with constant prior (left) and adaptive prior (right) is compared with the exact solution as well as for a Lagrange parameter leading to a weighted sum of squared residuals with $\chi_r^2 = 1$.

Finding fixed points efficiently There are several very efficient algorithms for increasing the convergence rate of contractive fixed point operators:

Lewitts method Lewitt et al. (Lewitt & Muehllehner, 1986) have suggested to increase the convergence rate by introducing an over-relaxation parameter in the iter-

ation loop:

$$x_j^{(k+1)} = x_j^{(k)} + \lambda^{(k)} \Delta x_j^{(k)} \tag{56}$$

where $\lambda^{(k)}$ is the over-relaxation parameter ($\lambda^{(k)} > 1$) whose purpose is to accelerate the iterative process, and whose value is not so large as to violate the non-negativity condition $x_j^{(k+1)} > 0$. To ensure that the over-relaxation of negative corrections to the solution vector does not decrease the value of any solution vector component below zero they suggest the following algorithm: first a pseudo-relaxation parameter $\mu_j^{(k)}$ is calculated where

$$\mu_j^{(k)} = \begin{cases} \infty, & \text{if } x_j^{(k)} \geq 0 \\ \left| \frac{x_j^{(k)}}{\Delta x_j^{(k)}} \right|, & \text{otherwise.} \end{cases} \tag{57}$$

Then they defined a critical relaxation parameter as follows:

$$\hat{\lambda}^{(k)} = \min_j \mu_j^{(k)} \tag{58}$$

Finally the over-relaxation parameter $\lambda^{(k)}$ was chosen according to

$$\lambda^{(k)} = \min\left[\left(\hat{\lambda}^{(k)} - 1\right)/2, \lambda^{\max}\right] \tag{59}$$

with $\lambda^{\max} \simeq 4$ so that $1 \leq \lambda^{(k)} \leq \lambda^{\max}$.

Anderson acceleration Another more efficient method to accelerate the convergence rate of the EM iteration scheme is the so called Anderson acceleration (Anderson, 1965) for solving fixed point problems [see also (Walker & Ni, 2011; Toth & Kelley, 2015)], it has also been suggested for the EM iteration scheme (Henderson & Varadhan, 2019). In SASfit several fixed point accelerations are supplied by making use of the sundials library (Hindmarsh *et al.*, 2005). However, only the Anderson acceleration speeds up the fixed point iterations in a reliable way whereas all the others

such as GMRES (Saad & Schultz, 1986), FGMRES (Saad, 1993), Bi-CGStab (van der Vorst, 1992) or TFQMR (Freund, 1993) become unstable and do not converge.

Biggs-Andrews method In (Biggs & Andrews, 1997; Biggs & Andrews, 1995; Biggs, 1998; Wang & Miller, 2014; Jiang *et al.*, 2017) an acceleration process has been suggested based on vector extrapolation which does not require an extra evaluation of the fixed point operator $\mathcal{O}_{\mathrm{EM}}$ in eq. 36. The method just needs to remember one or two previous solution vectors for calculating either the first or additionally the second derivative for guessing the next virtual solution vector, which is then used as an input vector for the next step of the fixed point iteration. The algorithm is described in the pseudo-code listing of fig. 14. The convergence behaviour of the different acceleration schemes are compared in fig. 15. Quite good acceleration has been achieved by the Biggs-Andrews method as well as by the Anderson acceleration, whereas other classical methods such as GMRES, Bi-CGSab or TFQMR failed. The Picard iteration scheme and over-relaxation method need orders of magnitudes more iterations to reach the true fixed point solution. The preferred acceleration scheme therefore becomes the Biggs-Andrews method.

procedure UPDATE_VECTORS($\mathbf{g}^{(n-2)}$,$\mathbf{g}^{(n-1)}$,$\mathbf{x}^{(n+1)}$,$\mathbf{x}^{(n)}$,$\mathbf{x}^{(n-1)}$,$\mathbf{x}^{(n-2)}$)
  $\mathbf{g}^{(n-2)} \leftarrow \mathbf{g}^{(n-1)}$
  $\mathbf{g}^{(n-1)} \leftarrow \mathbf{x}^{(n+1)} - \mathbf{x}^{(n)}$
  $\mathbf{x}^{(n-2)} \leftarrow \mathbf{x}^{(n-1)}$
  $\mathbf{x}^{(n-1)} \leftarrow \mathbf{x}^{(n)}$
  $\mathbf{x}^{(n)} \leftarrow \mathbf{x}^{(n+1)}$
end procedure
▷ Acceleration of the fixed point operator $\mathcal{O}[\mathbf{x}] = \mathbf{x}$
procedure BIGGS_ANDREWS_ACCELERATION($\mathcal{O}[]$, $\mathbf{x}^{(0)}$, o)
  $\alpha \leftarrow 0$
  $\mathbf{x}^{(n)} \leftarrow \mathbf{x}^{(0)}$
  $\mathbf{x}^{(n+1)} \leftarrow \mathcal{O}[\mathbf{x}^{(n)}]$
  UPDATE_VECTORS($\mathbf{g}^{(n-2)}$,$\mathbf{g}^{(n-1)}$,$\mathbf{x}^{(n+1)}$,$\mathbf{x}^{(n)}$,$\mathbf{x}^{(n-1)}$,$\mathbf{x}^{(n-2)}$)
  $\mathbf{x}^{(n+1)} \leftarrow \mathcal{O}[\mathbf{x}^{(n)}]$
  UPDATE_VECTORS($\mathbf{g}^{(n-2)}$,$\mathbf{g}^{(n-1)}$,$\mathbf{x}^{(n+1)}$,$\mathbf{x}^{(n)}$,$\mathbf{x}^{(n-1)}$,$\mathbf{x}^{(n-2)}$)
  $\mathbf{y}^{(n)} \leftarrow \mathbf{x}^{(n+1)}$
  while ($\mathcal{O}[]$ not converged) **do**
    ▷ α needs to be updated first
    $\alpha = \frac{\left(\mathbf{g}^{(n-1)}\right)^T \mathbf{g}^{(n-2)}}{\left(\mathbf{g}^{(n-2)}\right)^T \mathbf{g}^{(n-2)}}$
    $\alpha \leftarrow \max(\min(\alpha, 1), 0)$
    $\mathbf{x}^{(n+1)} \leftarrow \mathcal{O}[\mathbf{y}^{(n)}]$
    ▷ guess of next virtual step $\mathbf{y}^{(n)}$ calculated up to o^{th}-order
    if $\alpha = 0 \wedge o = 0$ **then**
      $\mathbf{y}^{(n)} \leftarrow \mathbf{x}^{(n)}$
    else if $\alpha > 0 \vee o = 1$ **then**
      $\mathbf{y}^{(n)} \leftarrow \mathbf{x}^{(n)} + \alpha \left(\mathbf{x}^{(n)} - \mathbf{x}^{(n-1)}\right)$
    else
      $\mathbf{y}^{(n)} \leftarrow \mathbf{x}^{(n)}\mathbf{x}^{(n)} + \alpha \left(\mathbf{x}^{(n)} - \mathbf{x}^{(n-1)}\right) + \frac{\alpha^2}{2} \left(\mathbf{x}^{(n)} + 2\mathbf{x}^{(n-1)} - \mathbf{x}^{(n-2)}\right)$
    end if
    ▷ now all the other temporary vectors are updated
    UPDATE_VECTORS($\mathbf{g}^{(n-2)}$,$\mathbf{g}^{(n-1)}$,$\mathbf{x}^{(n+1)}$,$\mathbf{x}^{(n)}$,$\mathbf{x}^{(n-1)}$,$\mathbf{x}^{(n-2)}$)
  end while
end procedure

Fig. 14. Pseudo-code for Biggs-Andrews vector extrapolation acceleration of order $o = 0, 1, 2$.

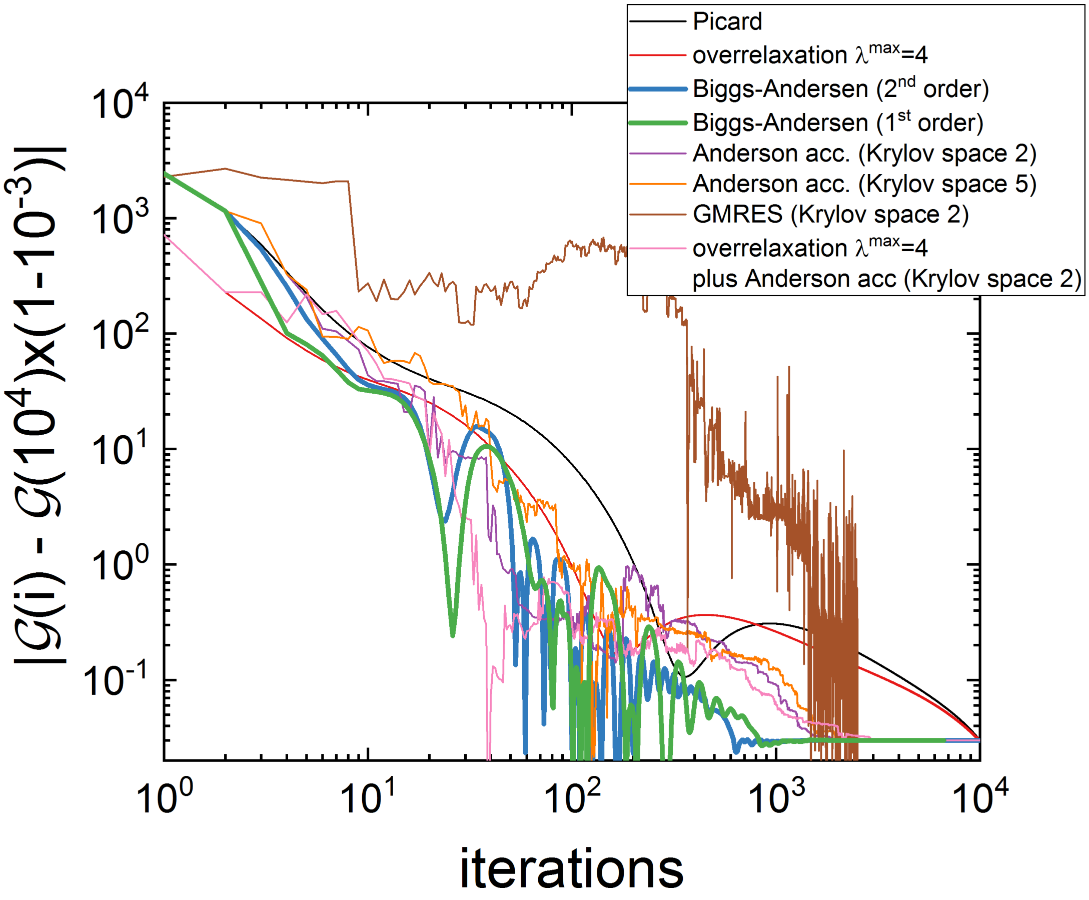

Fig. 15. Convergence behaviour of selected acceleration schemes compared to the non-accelerated Picard iteration.

3. Structure factors from solving Ornstein-Zernike equations

The central part of the SASfit algorithm to obtain a structure factor from a given pair interaction potential performs an iterative solution of the Ornstein-Zernike equation (Ornstein & Zernike, 1914). The theoretical background can be found in great detail in (Nägele, 2004; Borowko *et al.*, 2000; Hansen & McDonald, 2013; Bomont, 2008; Caccamo, 1996; Likos, 2001). The Ornstein-Zernike equation reads

$$h(\mathbf{r}_{12}) = c(\mathbf{r}_{12}) + \int c(\mathbf{r}_{13})\rho(\mathbf{r}_3)h(\mathbf{r}_{32})d\mathbf{r}_3 + \cdots \tag{60}$$

where $h(\mathbf{r}_{12})$ is the total correlation function, $c(\mathbf{r}_{12})$ the direct correlation function (direct effect of particle 1 on particle 2), $c(\mathbf{r}_{13})$ the direct correlation function describing the effect of particle 1 on particle 3 which influences particle 2, and ρ the particle number density of the colloids, molecules, or atoms that form the liquid. If the fluid is uniform and isotropic, the Ornstein–Zernike relation becomes

$$h(r) = c(r) + \rho \int c(|\mathbf{r} - \mathbf{r}'|)h(r')d\mathbf{r}' \tag{61}$$

$$= c(r) + \gamma(r) \tag{62}$$

The basic idea of this equation is that the total correlation between positions of two particles is a combination of their direct and indirect (through neighboring particles) interactions. The first contribution to $h(r)$ is the direct correlation function $c(r)$ that represents the correlation between a particle of a pair with its closest neighbor separated by a distance r. The second contribution is the indirect correlation function $\gamma(r)$, which represents the correlation between the selected particle of the pair with the rest of the fluid constituents.

$g(r)$ is known as the radial or pair distribution function, which measures the probability that given a particle at the origin, another particle of the fluid can be found at a distance r from it. When the distance separating a pair of particles tends to infinity, the correlations vanish and $g(r)$ tends to 1. This means that the total correlation function defined as $h(r) = g(r) - 1$ tends to 0. The Fourier transform $\mathcal{F}\{\}$ of $g(r)$ and $h(r)$ are directly related to the structure factor $S(q)$ that is experimentally measurable by x-ray or neutron scattering

$$S(q) = 1 + \rho \int [g(r) - 1] \exp(-\imath \mathbf{q} \cdot \mathbf{r})\, d\mathbf{r} \tag{63}$$

and

$$S(q) = 1 + \rho\tilde{h}(q) \tag{64}$$

where $\tilde{h}(q) = \mathcal{F}\{h(r)\}$ is the Fourier transform of the total correlation function $h(r)$, so that

$$\tilde{h}(q) = \int h(r) \exp\left(-\imath \mathbf{q}\cdot\mathbf{r}\right) d\mathbf{r} \tag{65}$$

Via inverse Fourier transform $\mathcal{F}^{-1}\{\}$ of the structure factor the pair correlation function is obtained

$$\rho\left[g(r) - 1\right] = \mathcal{F}^{-1}\left\{S(q) - 1\right\} = \frac{1}{(2\pi)^3}\int \left(S(q) - 1\right) \exp\left(\imath \mathbf{q}\cdot\mathbf{r}\right) d\mathbf{q} \tag{66}$$

As for $g(r)$ also the direct correlation function $c(r)$ can be, in principle, derived from the experiment as

$$\rho c(r) = \frac{1}{(2\pi)^3}\int \left(1 - \frac{1}{S(q)}\right) \exp\left(\imath \mathbf{q}\cdot\mathbf{r}\right) d\mathbf{q} \tag{67}$$

or

$$S(q) = \frac{1}{1 - \rho\tilde{c}(q)} \tag{68}$$

By Fourier transformation of eq. 61, one obtains

$$\tilde{h}(q) = \frac{\tilde{c}(q)}{1 - \rho\tilde{c}(q)} \tag{69}$$

where $\tilde{c}(q) = \mathcal{F}\{c(r)\}$ is the Fourier Transform of $c(r)$. In order to determine the two correlation functions $h(r)$ and $c(r)$ for a given pair potential $u(r)$, eq. 61 must be

supplemented by an auxiliary closure relation. Furthermore the Fourier transform of eq. 62 in combination with eq. 69 allows to write the Fourier transform of the indirect correlation function as

$$\mathcal{F}\{\gamma(r)\} = \tilde{\gamma}(q) = \tilde{h}(q) - \tilde{c}(q) = \frac{\rho\tilde{c}^2(q)}{1-\rho\tilde{c}(q)} \tag{70}$$

The closure is not complete as long the bridge function is unknown. Even though the bridge function is exactly defined the function is an unknown function of inter-particle distance and only approximations can be given. The direct correlation function can be written in terms of the indirect correlation function, the interaction potential and the bridge function as

$$\begin{aligned} c(r) &= \exp\left[-\beta u(r) + \gamma(r) + B(r)\right] - \gamma(r) - 1 \qquad (71) \\ &= g(r) - \gamma(r) - 1 \\ \text{and } h(r) + 1 = g(r) &= \exp\left[-\beta u(r) + \gamma(r) + B(r)\right] \qquad (72) \end{aligned}$$

where $u(r)$ is the pair interaction potential, $\beta = 1/(k_B T)$ the inverse temperature, with k_B being Boltzmann's constant, T is the absolute temperature, $\gamma(r)$ being the indirect correlation function and ρ is the number density of the molecules or atoms that form the liquid. $B(r)$ is the bridge function. The analytic expression of $B(r)$ is, in general, unknown. Unfortunately, the bridge function has to be approximated in practice. Though a number of theoretical and simulation procedures have been derived in recent years in order to get an approximate estimate of $B(r)$ for different fluid models, the exact bridge function is not known for any system.

The easiest algorithm for solving the Ornstein Zernike (OZ) equation 61 together with the closure equation 71 is direct iteration using fast Fourier transformation [see e.g. (Homeier *et al.*, 1995)]. The solution of the OZ equations can be treated either

as a fixed point problem for the unknown function $\gamma(r)$, so that $T_f^{\mathrm{OZ}}(\gamma) = \gamma$ or as a multidimensional root finding problem $F_r^{\mathrm{OZ}}(\gamma) = T_f^{\mathrm{OZ}}(\gamma) - \gamma = 0$. In SASfit the fixed point problem $T_f^{\mathrm{OZ}}(\gamma) = \gamma$ is implemented as:

1. Set a starting value for $\gamma(r)$, e.q. set $\gamma(r) \equiv 0$
2. Calculate a bridge function. Many different theories how to calculate the bridge function have been published, where the HNC, PY, RMSA, HMSA, RY are the most used ones. The bridge function is often expressed in terms of the known potential $u(r)$ and the indirect correlation function $\gamma(r)$ which needs to be determined.
3. Calculate the direct correlation function according to eq. 71.
4. Calculate the Fourier transform $\tilde{c}(q)$ of the direct correlation function.
5. Use eq. 70 to get $\tilde{\gamma}(q)$.
6. Calculate the inverse Fourier transform of $\tilde{\gamma}(q)$ to get the new guess for $\gamma(r)$.

This algorithm for solving the Ornstein-Zernike equation iteratively has to perform two Fourier transforms:

$$c(r) \xrightarrow{\mathcal{F}\{\}} \tilde{c}(q) \quad \text{(in step 4)} \tag{73}$$

$$\tilde{\gamma}(q) \xrightarrow{\mathcal{F}^{-1}\{\}} \gamma(r) \quad \text{(in step 6)} \tag{74}$$

As we assume that the system is isotropic, i.e. $c(r)$ and $\gamma(r)$ are only functions of the modulus of $|\mathbf{r}| = r$, the Fourier transforms can be written as

$$\tilde{c}(q) = \int c(r) \exp\left(-\imath \mathbf{q} \cdot \mathbf{r}\right) d\mathbf{r} = \int_0^\infty 4\pi r^2 c(r) \frac{\sin(qr)}{qr} dr \tag{75}$$

$$\gamma(r) = \frac{1}{(2\pi)^3} \int \tilde{\gamma}(q) \exp\left(\imath \mathbf{q} \cdot \mathbf{r}\right) d\mathbf{q} = \frac{1}{(2\pi)^3} \int_0^\infty 4\pi q^2 \tilde{\gamma}(q) \frac{\sin(qr)}{qr} dq \tag{76}$$

To calculate these transforms numerically on a discrete grid of points, the integration has to be substituted by a summation. Hereby, $\int_0^\infty dr$ is replaced by $\sum_{j=0}^{N_p-1} \Delta r$, $r_j = (j+1)\Delta r$, $q_j = (j+1)\Delta q$, and $\int_0^\infty dq$ is replaced by $\sum_{j=0}^{N_p-1} \Delta q$. The step width Δr in real space has to be defined by the user as well as the number of grid points N_p. The step width in reciprocal space is than obtained by $\Delta q = \frac{\pi}{(N_p+1)\Delta r}$. It is important to mention that the values of the first elements of the arrays r_i and k_i equal Δr and Δq, respectively $(r_0 = \Delta r, q_0 = \Delta q)$:

$$\tilde{c}_i = \frac{4\pi\Delta r^2}{(i+1)\Delta q} \sum_{j=0}^{N_p-1} c_j (j+1) \sin\left(\frac{\pi(j+1)(i+1)}{N_p+1}\right) \tag{77}$$

$$\gamma_i = \frac{1}{(2\pi)^3} \frac{4\pi\Delta q^2}{(i+1)\Delta r} \sum_{j=0}^{N_p-1} \tilde{\gamma}_j (j+1) \sin\left(\frac{\pi(j+1)(i+1)}{N_p+1}\right) \tag{78}$$

For calculating the Fourier transformation the library `FFTW` for fast Fourier transformation (Frigo & Johnson, 2005; Frigo & Johnson, 1997) has been used (source code available at `http://www.fftw.org`). The library supplies a discrete sin-transformation (type-I DST) named `FFTW_RODFT00` which is doing the transformation:

$$Y_k = 2 \sum_{j=0}^{N_p-1} X_j \sin\left(\frac{\pi(j+1)(k+1)}{N_p+1}\right) \tag{79}$$

The unnormalized inverse of `FFTW_RODFT00` is `FFTW_RODFT00` itself. The same routine can be used for both transformations.

For the fixed point problem $T_f^{\mathrm{OZ}}(\gamma) = \gamma$ several iteration schemes are implemented. Tests have shown, that no prediction can be made which one converges fastest or in case of e.g. very high volume or strong attraction, converges at all. Next to some simple iteration schemes also a the very efficient Anderson Acceleration for fixed-point iteration (Anderson, 1965; Walker & Ni, 2011; Toth & Kelley, 2015) has been implemented. Besides these algorithms also several very efficient nonlinear system

solvers based on Newton-Krylov solver technology, like GMRES (Generalized Minimal RESidual) (Saad & Schultz, 1986; Kelley, 2003), FGMRES (Flexible Generalized Minimal RESidual) (Saad, 1993), Bi-CGStab (Bi-Conjugate Gradient Stabilized) (van der Vorst, 1992; Kelley, 2003), and TFQMR (Transpose-Free Quasi-Minimal Residual) (Freund, 1993; Kelley, 2003) are supplied. These nonlinear system solvers are made available through the 'kinsol' library which is part of the SUNDIALS package (Hindmarsh *et al.*, 2005) available at `https://computing.llnl.gov/projects/sundials`. Test have shown that either the Anderson acceleration, GMRES or FGMRES seems to be the most efficient algorithm to solve the Ornstein-Zernike equations with a minimum number of calls to the procedure evaluating the self consistent operator $F_r^{\mathrm{OZ}}(\gamma)$ or $T_f^{\mathrm{OZ}}(\gamma)$. The best choice for the iteration method depends often on the problem, i.e. the type of closure and the potential and also on the volume fraction. Therefore, all methods mentioned above are still available in the program which might change after getting some more experience with them.

4. Parameters of numerical algorithms

Many numerical algorithms and their parameters in SASfit can be adjusted in detail if the particular application requires it. For example, the more efficient and flexible routine `sasfit_cubature` for numerical integrations in multiple dimensions and a routine for optimized spherical averages (`sasfit_orient_avg`) was implemented. For both routines, an algorithm can be chosen by the user in a dedicated menu shown in figure 16 along with the respective parameters. Each integration strategy is also supplied as a multiple integration routine. Several of them are just nested calls and only few are designed for multidimensional integration. Because the menu interface should reflect this better, its revision is planned for the future.

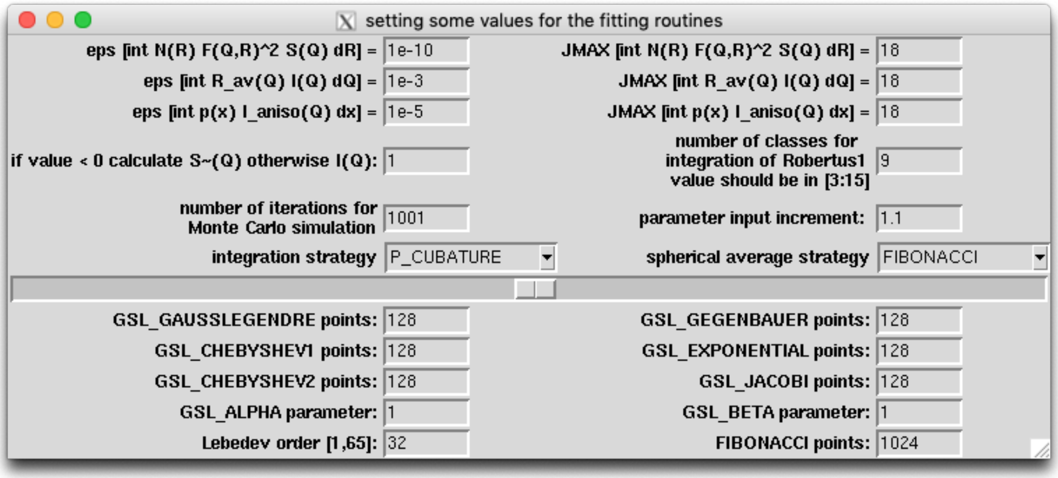

Fig. 16. A dedicated menu for choosing a numerical integration routine with its respective parameters especially for multi-dimensional integrations (`sasfit_cubature` routine) and spherical averages (`sasfit_orient_avg` routine). This menu can be found in the fit or simulation window menu bar under '*Options*' → '*Customize*'.

References

Anderson, D. G. (1965). *Journal of the ACM*, **12**(4), 547–560.

Arslan, K., Bulca, B., Bayram, B., Ozturk, G. & Ugail, H. (2009). In *2009 International Conference on CyberWorlds*. IEEE.

Bakry, M., Haddar, H. & Bunău, O. (2019). *Journal of Applied Crystallography*, **52**(5).

Barr (1981). *IEEE Computer Graphics and Applications*, **1**(1), 11–23.

Benvenuto, F. (2017). *SIAM Journal on Numerical Analysis*, **55**(5), 2187–2203.

Benvenuto, F., Haddar, H. & Lantz, B. (2016). *SIAM Journal on Applied Mathematics*, **76**(1), 276–292. Cited By 0.

Biggs, D. S. (1998). *Accelerated iterative blind deconvolution*. Ph.D. thesis, The University of Auckland.
http://hdl.handle.net/2292/1760

Biggs, D. S. C. & Andrews, M. (1995). *Electronics Letters*, **31**(23), 1985–1986.

Biggs, D. S. C. & Andrews, M. (1997). *Applied Optics*, **36**(8), 1766.

Blanc, C. & Schlick, C. (1996). *The Visual Computer*, **12**(8), 420–428.

Bomont, J.-M. (2008). *Recent Advances in the Field of Integral Equation Theories: Bridge Functions and Applications to Classical Fluids*, chap. Chapter 1, pp. 1–84. John Wiley & Sons, Inc.

Borowko, M., Sokolowski, S. & Henderson, D. (2000). In *Surfactant Science*, chap. 3, pp. 135–165. CRC Press.

Breßler, I., Kohlbrecher, J. & Thünemann, A. F. (2015). *Journal of Applied Crystallography*, **48**(5), 1587–1598.

Byrne, C. & Eggermont, P. P. B. (2011). In *Handbook of Mathematical Methods in Imaging*, pp. 271–344. Springer New York.

Caccamo, C. (1996). *Physics Reports*, **274**(1-2), 1–105.

Chae, M., Martin, R. & Walker, S. G. (2018). *Statistics and Computing*, **29**(4), 645–654.

Dempster, A. P., Laird, N. M. & Rubin, D. B. (1977). *Journal of the Royal Statistical Society. Series B (Methodological)*, **39**(1), 1–38.

Donatelli, M. & Reichel, L. (2014). *Journal of Computational and Applied Mathematics*, **272**, 334–349.

Eggermont, P. P. B. (1999). *Applied Mathematics and Optimization*, **39**(1), 75–91.

Eggermont, P. P. B. & LaRiccia, V. N. (1995). *The Annals of Statistics*, **23**(1), 199–220.

Fougerolle, Y., Gribok, A., Foufou, S., Truchetet, F. & Abidi, M. (2005*a*). *IEEE Transactions on Visualization and Computer Graphics*, **11**(5), 529–539.

Fougerolle, Y., Gribok, A., Foufou, S., Truchetet, F. & Abidi, M. (2005*b*). In *In Proc. of Pacific Graphics*, pp. 169–172.

Fougerolle, Y. D., Gribok, A., Foufou, S., Truchetet, F. & Abidi, M. A. (2006). *Journal of Computer Science and Technology*, **21**(2), 238–243.

Fougerolle, Y. D., Gribok, A., Foufou, S., Truchetet, F. & Abidi, M. A. (2007). In *Eighth International Conference on Quality Control by Artificial Vision*, edited by D. Fofi & F. Meriaudeau. SPIE.

Freund, R. W. (1993). *SIAM Journal on Scientific Computing*, **14**(2), 470–482.

Frigo, M. & Johnson, S. G. (1997). *The Fastest Fourier Transform in the West*. Tech. Rep. MIT-LCS-TR-728. .
http://www.fftw.org/fftw-paper.pdf

Frigo, M. & Johnson, S. G. (2005). *Proceedings of the IEEE*, **93**(2), 216–231. Special issue on "Program Generation, Optimization, and Platform Adaptation".

Gielis, J. (2003). *American Journal of Botany*, **90**(3), 333–338.

Glatter, O. (1977). *Journal of Applied Crystallography*, **10**(5), 415–421.

Hansen, J.-P. & McDonald, I. R. (2013). *Theory of Simple Liquids with Applications to Soft Matter*. Academic Press, 4th ed.

Hansen, P. (2000). In *Computational Inverse Problems in Electrocardiology*. WIT Press.

Hansen, P. C. & O'Leary, D. P. (1993). *SIAM Journal on Scientific Computing*, **14**(6), 1487–1503.

Hansen, S. & Müller, J. J. (1996). *Maximum Entropy and Bayesian Methods*, chap. The Maximum-Entropy Method in Small-Angle Scattering, pp. 69–78. Dordrecht: Springer Netherlands.

Hansen, S. & Pedersen, J. S. (1991). *Journal of Applied Crystallography*, **24**(5), 541–548.

Henderson, N. C. & Varadhan, R. (2019). *Journal of Computational and Graphical Statistics*, pp. 1–13.

Hindmarsh, A. C., Brown, P. N., Grant, K. E., Lee, S. L., Serban, R., Shumaker, D. E. & Woodward, C. S. (2005). *ACM Transactions on Mathematical Software*, **31**(3), 363–396.

Homeier, H. H., Rast, S. & Krienke, H. (1995). *Computer Physics Communications*, **92**(2-3), 188–202.

Horne, K. (1985). *Monthly Notices of the Royal Astronomical Society*, **213**(2), 129–141.

Jiang, J., Huang, J. & Zhang, G. (2017). *IEEE Sensors Journal*, **17**(5), 1306–1315.

Johnson, S. G., (2020). C package for adaptive multidimensional integration (cubature).
https://github.com/stevengj/cubature

Keelin, T. W. (2016). *Decision Analysis*, **13**(4), 243–277.

Keelin, T. W., Chrisman, L. & Savage, S. L. (2019). *The Metalog Distributions and Extremely Accurate Sums of Lognormals in Closed Form*, pp. 3074–3085. IEEE Press.

Keelin, T. W. & Powley, B. W. (2011). *Decision Analysis*, **8**(3), 206–219.

Kelley, C. T. (2003). *Solving Nonlinear Equations with Newton's Method*. Society for Industrial and Applied Mathematics.

Kullback, S. & Leibler, R. A. (1951). *The Annals of Mathematical Statistics*, **22**(1), 79–86.

Lebedev, V. (1975). *USSR Computational Mathematics and Mathematical Physics*, **15**(1), 44–51.

Lebedev, V. (1976). *USSR Computational Mathematics and Mathematical Physics*, **16**(2), 10–24.

Lebedev, V. I. (1977). *Siberian Mathematical Journal*, **18**(1), 99–107.

Lewitt, R. M. & Muehllehner, G. (1986). *IEEE Transactions on Medical Imaging*, **5**(1), 16–22.

Likos, C. N. (2001). *Physics Reports*, **348**(4-5), 267–439.

Lucy, L. B. (1974). *The Astronomical Journal*, **79**, 745.

Lucy, L. B. (1994). *Astronomy and Astrophysics*, **289**, 983–994.

Marques, R., Bouville, C., Ribardière, M., Santos, L. P. & Bouatouch, K. (2013). *Computer Graphics Forum*, **32**(8), 134–143.

Nägele, G., (2004). The physics of colloidal soft matter.

Niederreiter, H. (1992).

Ornstein, L. & Zernike, F. (1914). *Koninklijke Nederlandsche Akademie van Wetenschappen Proceedings*, **17**, 793–806.

Pedersen, J. S. (1997). *Advances in Colloid and Interface Science*, **70**(0), 171–210.

Richardson, W. H. (1972). *Journal of the Optical Society of America*, **62**(1), 55.

Saad, Y. (1993). *SIAM Journal on Scientific Computing*, **14**(2), 461–469.

Saad, Y. & Schultz, M. H. (1986). *SIAM Journal on Scientific and Statistical Computing*, **7**(3), 856–869.

Steenstrup, S. & Hansen, S. (1994). *Journal of Applied Crystallography*, **27**(4), 574–580.

Svergun, D. I. (1992). *Journal of Applied Crystallography*, **25**(4), 495–503.

Svergun, D. I., Semenyuk, A. V. & Feigin, L. A. (1988). *Acta Crystallographica Section A*, **44**(3), 244–250.

Tikhonov, A. N. (1943). *Doklady Akademii Nauk SSSR*, **39**, 195–198.

Tikhonov, A. N., Goncharsky, A. V., Stepanov, V. V. & Yagola, A. G. (1995). *Numerical Methods for the Solution of Ill-Posed Problems*. Springer Netherlands.

Toth, A. & Kelley, C. T. (2015). *SIAM Journal on Numerical Analysis*, **53**(2), 805–819.

Vardi, Y. & Lee, D. (1993). *Journal of the Royal Statistical Society: Series B (Methodological)*, **55**(3), 569–598.

van der Vorst, H. A. (1992). *SIAM Journal on Scientific and Statistical Computing*, **13**(2), 631–644.

Walker, H. F. & Ni, P. (2011). *SIAM Journal on Numerical Analysis*, **49**(4), 1715–1735.

Wang, H. & Miller, P. C. (2014). *IEEE Transactions on Image Processing*, **23**(2), 848–854.

Yang, F., Wang, A., Dong, L. & Ming, H. (2013). *Optical Engineering*, **52**(4), 043605.
